# Altered Network Topologies and Hub Organization in Adults with Autism: A Resting-State fMRI Study

**DOI:** 10.1371/journal.pone.0094115

**Published:** 2014-04-08

**Authors:** Takashi Itahashi, Takashi Yamada, Hiromi Watanabe, Motoaki Nakamura, Daiki Jimbo, Seiji Shioda, Kazuo Toriizuka, Nobumasa Kato, Ryuichiro Hashimoto

**Affiliations:** 1 Department of Pharmacognosy and Phytochemistry, Showa University School of Pharmacy, Tokyo, Japan; 2 Department of Psychiatry, Showa University School of Medicine, Tokyo, Japan; 3 Kinko Hospital, Kanagawa Psychiatric Center, Kanagawa, Japan; 4 Department of Anatomy, Showa University School of Medicine, Tokyo, Japan; 5 Department of Language Sciences, Graduate School of Humanities, Tokyo Metropolitan University, Tokyo, Japan; Wake Forest School of Medicine, United States of America

## Abstract

Recent functional magnetic resonance imaging (fMRI) studies on autism spectrum condition (ASC) have identified dysfunctions in specific brain networks involved in social and non-social cognition that persist into adulthood. Although increasing numbers of fMRI studies have revealed atypical functional connectivity in the adult ASC brain, such functional alterations at the network level have not yet been fully characterized within the recently developed graph-theoretical framework. Here, we applied a graph-theoretical analysis to resting-state fMRI data acquired from 46 adults with ASC and 46 age- and gender-matched controls, to investigate the topological properties and organization of autistic brain network. Analyses of global metrics revealed that, relative to the controls, participants with ASC exhibited significant decreases in clustering coefficient and characteristic path length, indicating a shift towards randomized organization. Furthermore, analyses of local metrics revealed a significantly altered organization of the hub nodes in ASC, as shown by analyses of hub disruption indices using multiple local metrics and by a loss of “hubness” in several nodes (e.g., the bilateral superior temporal sulcus, right dorsolateral prefrontal cortex, and precuneus) that are critical for social and non-social cognitive functions. In particular, local metrics of the anterior cingulate cortex consistently showed significant negative correlations with the Autism-Spectrum Quotient score. Our results demonstrate altered patterns of global and local topological properties that may underlie impaired social and non-social cognition in ASC.

## Introduction

Autism spectrum condition (ASC) has been characterized by impairments in social and communication skills, combined with repetitive and restricted behavior. However, this spectrum comprises a range of heterogeneous populations with varying degrees of severity. The debates surrounding the precise definition of its core symptoms, as well as the choice of the term for characterizing this spectrum (i.e., “disorder” or “condition”), have not been settled even in the latest version of the American Psychiatry Association’s Diagnostic and Statistical Manual of Mental Disorders-Fifth Edition (DSM-V) has been published [1]. In an effort to reveal functional brain alterations underlying the clinical symptoms of ASC, an increasing number of functional magnetic resonance imaging (fMRI) studies have recently examined intrinsic activity in the absence of externally imposed tasks. Such resting-state fMRI (rs-fMRI) studies have found atypical intrinsic connectivity in ASC (for overview, see [2]): for instance, under-connectivity within a default mode network [3], [4], and over- and under-connectivity between many pairs of brain regions [5], [6]. These abnormal functional interactions between distributed brain areas lend support to the hypothesis that brain abnormalities in ASC can best be understood as a network disorder [7].

In neuroscience research, network studies (connectomics) of the human brain are becoming the key to understanding brain functions and mechanisms of neuropathological disorders [8]. In particular, advances in graph-theoretical analysis of rs-fMRI data have provided a new perspective on the functional organization of the human brain. This approach enables the representation of the whole brain as a large-scale network composed of nodes (brain regions) and edges (connections between nodes), and allows the examination of the topological properties of the network [9]. At the network level, for example, a small-world network is characterized by high clustering and short characteristic path length [10], which is a hallmark of the human brain network [11], [12]. At the nodal level, the degree of a node quantifies the connectedness of the node with the rest of nodes in a network and therefore is useful in identifying highly connected nodes (i.e., hubs) that may play critical roles in information integration [13]. Several studies have identified functional hubs, particularly along the midline cortical regions (e.g., the supplementary motor area and precuneus) in neurotypical individuals [14]–[17]. While network structure and topological metrics change during the normal course of development [18]–[21], hub organization is stable from adolescence to young adulthood in neurotypical individuals [17], providing the notion that hub organization may serve as a functional backbone of the human brain. On the other hand, there is evidence for atypical neurodevelopment during adolescence in individuals with ASC [22], which raises the possibility that significant changes in hub organization occur between adolescence and adulthood.

A number of studies have applied graph-theoretical analysis to clinical populations, including patients with schizophrenia [23]–[27], major depressive disorder [28], and other disorders [29], [30]. Although the graph-theoretical approaches are now being applied to studies on different aspects of the ASC brain (e.g., white matter connectivity and electrophysiological connectivity) [31]–[35], only a few have applied graph-theoretical analysis to rs-fMRI data of this disorder [36]–[39]. Rudie et al. [39] recently described disrupted local segregation and enhanced global integration in adolescents with ASC compared to neurotypical counterparts, while Barttfeld et al. [36] have reported that changes in the small-worldness measure occurring at shifts between different cognitive states show opposite patterns in patients with ASC and controls. However, basic questions regarding global network organization and possible alterations of “hubness” in adults with ASC have not been fully addressed.

Growing evidence suggests that people with ASC exhibit atypical neurodevelopmental process at least persisting immediately before adulthood [22], [40]–[42]. For instance, individuals with ASC show brain over-growth in early childhood, followed by a period with an accelerated rate of decline in brain size from adolescence to middle age [42]. In addition, the developmental trajectories of subcortical regions (e.g., putamen and caudate) are known to be different in individuals with ASC relative to normal controls [43]. Furthermore, functional abnormalities of ASC vary with age [44], as corroborated by a meta-analysis revealing that the neural anomalies in adults with ASC differed significantly from those in children [45]. Therefore, in addition to the previous pediatric data, it is important to investigate the topological properties of the brain network in adults with ASC to complement the time-course of changes in topological properties from adolescence to adulthood.

The present study applied a graph-theoretical analysis to rs-fMRI data and compared the local and global topological properties of the brain network of adults with ASC to those of age- and gender-matched controls. In particular, we focused on the analysis of hub organization using local metrics and examined whether the order of node importance is altered in the autistic brain network. Since previous findings suggested that adults with ASC showed under-connectivity when compared to normal controls [2], we hypothesized that: 1) adults with ASC would show aberrant network organization as quantified by several global metrics, such as decreases in clustering coefficient and characteristic path length; 2) local network metrics would be altered in several regions for which activation and/or connectivity have been shown to be abnormal in ASC (e.g., the anterior cingulate cortex); 3) several topological properties altered in ASC would be linked with the autistic trait as assessed by the Autism-Spectrum Quotient (AQ) test [46]; and 4) given the altered pattern of behavioral strengths and weaknesses in ASC, participants with ASC would exhibit significantly altered hub organization and alterations of “hubness” in nodes (e.g., superior temporal sulcus) responsible for a broad range of dysfunctions in ASC.

## Materials and Methods

### 1. Participants

Forty-six adults with ASC were recruited from outpatient units of the Karasuyama Hospital, Tokyo, Japan. The inclusion criteria were: (1) age between 18 and 55 years and (2) a formal diagnosis of pervasive developmental disorder (PDD) based on the Diagnostic and Statistical Manual of Mental Disorders, Fourth Edition (DSM-IV). Exclusion criteria included a history of electroconvulsive therapy, alcohol or other drug abuse or dependence, or any neurological illness affecting the central nervous system. PDD was diagnosed by a team of three experienced psychiatrists and one clinical psychologist, based on two detailed interviews with the patients regarding their development and behavior from infancy through adolescence and family history. The interviews were conducted independently by one of the psychiatrists and the clinical psychologist in the team. The patients were also asked to bring along suitable informants who had known them in early childhood. The psychiatrist gave the final diagnosis, after consultation with the other psychiatrist and the clinical psychologist, a process requiring approximately three hours. This team confirmed that none of the participants met the DSM-IV criteria for any other psychiatric disorder. Although there were some individuals with ASC who showed some levels of anxiety, mood, or attention deficit and hyperactivity disorders, the severity of the problems did not meet the diagnostic criteria for other mental disorders.

A total of 46 normal controls (NCs) were recruited by advertisements and acquaintances. None of the NCs reported any severe medical problem or history of any neurological or psychiatric problems. Moreover, the Mini-International Neuropsychiatric Interview was used to confirm that none of the NCs met the diagnostic criteria for any psychiatric disorder.

The intelligence quotient (IQ) scores of participants with ASC were evaluated using either the Wechsler Adult Intelligence Scale-Third Edition (WAIS-III) or the WAIS-Revised (WAIS-R), while those of NCs were estimated using a Japanese version of the National Adult Reading Test (JART) [47]. Based on the National Adult Reading Test (NART) for English-speaking population [48], the JART has been developed to estimate the IQ of a Japanese subject by scoring his or her reading ability of 25 words printed in Kanji (adopted logographic Chinese characters). It has been widely used in Japanese clinical studies both for normal and patient groups [49], [50]. Every participant with ASC was considered as being high-functioning, because his or her full-scale IQ score was higher than 80. Although the IQ score was missing for one male participant with ASC, he was also regarded as being high-functioning, because his predicted IQ was 114 based on the JART. Handedness was assessed using the Edinburgh Handedness Inventory [51]. Furthermore, participants completed the Japanese version of the Autism-Spectrum Quotient (AQ) test [46], [52]. All of the participants of this study had normal or corrected-to-normal vision. Within the ASC group, 14 of the 46 participants were using either one or more of the following medications: antidepressants (9 patients), hypnotic drugs (9 patients), anti-anxiety drugs (8 patients), antipsychotic drugs (6 patients), and antiepileptic drugs (3 patients). The summary of participant demographic information can be found in Table 1.

**Table 1:** Demographics and rating scale of the participants.

	NC			ASC			Statistics	
	Mean ± s.d.	Range	*N*	Mean ± s.d.	Range	*N*	*df*	*p*-value
Sample size			46 (7 female)			46 (7 female)		
Age (years)	32.02±7.94	19–50	46	31.11±8.14	19–51	46	90	0.68
Handedness	80.68±49.40	-100–100	41	69.49±63.59	−100–100	45	84	0.37
Estimated IQ	107.59±8.64	87.46–119.8	44	105.8±14.12	83–134	45	87	0.47
AQ score	15.08±5.27	8–30	38	36.20±5.63	24–47	46	82	<0.001

NC: normal control, ASC: autism spectrum condition, s.d.: standard deviation, *N*: the sample size for each of demographic information, AQ: Autism Spectrum Quotient.

The Ethics Committee of the Faculty of Medicine of Showa University approved all of the procedures used in this study, including the method of obtaining consent, in accordance with the Declaration of Helsinki. Written informed consent was obtained from participants after fully explaining the purpose of this study. Since all participants were high-functioning (IQ>80) adults without any other comorbidities, they were able to fully understand the content and nature of this study. Guardians’ verbal consent was neither documented nor recorded because every patient was judged to possess the full ability to give consent on his or her own by his or her primary doctor (TY, WH, MN, or NK). Any concern regarding the possibility of reduced capacity to consent on his or her own was not voiced by either the ethics committee or patients’ primary doctors. Every participant was assigned an arbitrary identification number for this study, so that all the data, including imaging and demographic data, could be analyzed anonymously. The data were stored locally, at a single location in the Department of Psychiatry, Showa University. In accordance with the obtained written informed consent, the data were only available for use by our research group.

### 2. MRI Data Acquisition

All MRI data were acquired using a 1.5-Tesla GE Signa system (General Electric, Milwaukee, WI, USA) with a phased-array whole-head coil. The functional images were acquired using a gradient echo-planar imaging sequence (in-plane resolution: 3.4375×3.4375 mm, echo time (TE): 40 ms, repetition time (TR): 2000 ms, flip angle: 90°, slice thickness: 4 mm with a 1-mm slice gap [11], [53], matrix size: 64×64, 27 axial slices). Two hundred and eight volumes were acquired in a single run. The first four volumes were discarded to allow for T1 equilibration. In addition, a high-resolution T1-weighted spoiled gradient recalled (SPGR) 3D MRI image was collected (in-plane resolution: 0.9375×0.9375 mm, 1.4 mm slice thickness, TR: 25 ms, TE: 9.2 ms, matrix size: 256×256, 128 sagittal slices). Each participant was instructed to lie relaxed in the scanner and to remain as still as possible with his or her eyes closed, yet to stay awake in the dim scanner room.

### 3. Data Preprocessing

SPM8 software (http://www.fil.ion.ucl.ac.uk/spm/software/spm8/) was used to perform fMRI data preprocessing. First, slice timing and head motion were corrected. No participant was excluded due to excessive motion (>±2 mm translation and >±2° rotation from the first volume in any axis). We also evaluated the amount of translation and rotation of the head during the scanning of each subject according to the following equation [54]:
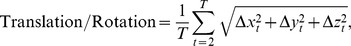
where *T* is the number of volumes (i.e., *T* = 204 in this study); variables *x*, *y,* and *z* stand for translation or rotation values in each of the three axes; and Δ*x_i_* is the difference between *x_i_* and *x_i-1_* in the *x*-axis. The amount of head motion was comparable between the two groups in both translation (NC: 0.049±0.026 (mean ± standard deviation); ASC: 0.043±0.024; *t*-test: *t* = 1.140, *p* = 0.257) and rotation (NC: 0.037±0.014; ASC: 0.034±0.014; *t*-test: *t* = 1.016, *p* = 0.312). For each participant, a T1-weighted SPGR image was realigned along the mid-sagittal anterior-posterior commissure line, and then the realigned T1-weighted image was segmented and reconstructed in order to generate a skull-tripped T1-weighted image. The realigned fMRI images were co-registered to the skull-stripped T1-weighted image; the fMRI images were then spatially normalized to the standard Montreal Neurological Institute (MNI) template, and were resampled to a resolution of 3×3×3 mm. Finally, the images were spatially smoothed using a 6-mm full-width half-maximum Gaussian kernel.

### 4. Network Construction

#### 4.1. Definitions of nodes and edges

Functional brain networks consist of nodes and edges. In this study, nodes and edges corresponded to regions of interest (ROIs) and functional connectivity between all possible pairs of the ROIs, respectively. We used the MNI coordinates of 160 brain regions provided by a previous meta-analytic study [55], and constructed a ROI consisting of voxels within a 5-mm radius sphere around the coordinate for each node. The mean time-series was extracted from each ROI, and then artifactual components were removed from the mean time-series using the CompCor method [56] implemented in the Functional Connectivity Toolbox (http://www.nitrc.org/projects/conn/). Briefly, this method first identifies five principal components associated with physiological signals from the segmented white matter and cerebrospinal fluid regions in each participant, and then regresses out those time-series, together with those associated with six head motion parameters and their temporal derivatives from the extracted mean time-series in each ROI. In this study, no global signal regression was performed to avoid the risk of yielding spurious negative correlations [57].

To reduce systematic biases in functional connectivity induced by sub-millimeter head motions during the scan [58], [59], we adopted the “scrubbing” method together with a frame-wise displacement (FD) threshold of 0.5 mm and a band-pass filter (0.009–0.08 Hz) [58] (see Text S1 and Figure S1 for details and the effect of scrubbing). We confirmed that the mean FD was comparable between the two groups (NC: 0.1153±0.0423; ASC: 0.1034±0.0466; *t* = 1.28, *p* = 0.201). After these scrubbing steps, the number of retained volumes was comparable between the groups (NC: 203.17±1.51 volumes; ASC; 202.93±2.51 volumes; *t*-test: *t* = 0.554, *p* = 0.581), and each group retained approximately 99.5% of their original volumes. For measuring functional connectivity, correlation coefficients between all possible pairs of the ROIs were then calculated, which resulted in a 160×160 correlation matrix for each participant. Finally, each correlation matrix was transformed into an adjacency matrix by applying a predefined threshold (see next section), where the *i*-th and the *j*-th nodes were connected if the (*i*,*j*)-th element of the adjacency matrix is equal to one. See Text S2 for functional connectivity analysis.

#### 4.2. Threshold selection

A binary undirected graph was constructed by applying a correlation threshold, ranging from zero to one, to each element of the correlation matrix; however, applying a single common threshold across all participants yields a different number of edges and nodes, which in turn may induce spurious between-group differences in network topology [60]. To avoid this problem, the sparsity-based threshold, *S*, the ratio of the number of existing edges to the maximum possible number of edges in a graph ( = 12720), was employed [26], [28].

The range of sparsity-based thresholds *S* was determined using the following procedures. First, we identified the minimum number of positive elements in the correlation matrix to determine the maximum sparsity (i.e., higher limit) for each participant, and then identified the minimum sparsity (i.e., lower limit), in which there was no fragmentation of the graph into several components for each participant. All graphs satisfied the property of the small-world network (i.e., the small-worldness scalar σ is greater than one). See the following section for a description of the small-worldness scalar. The range of sparsity was determined as 19.50% ≤ *S* ≤48.43%. Over this range, we repeatedly calculated the global and local network metrics of interest (see next section) with an interval of 0.63% (47 steps).

### 5. Network Metrics

To assess topological properties of graphs, local and global metrics were calculated on a graph at each of the 47 sparsity levels. Brief descriptions of the metrics used in this study are listed in Table S1. More detailed definitions and descriptions of the metrics can be found in a recent review [61]. For each node, we calculated the three local metrics: degree *k*, betweenness *b*, and nodal efficiency *e*. For global metrics, we calculated global efficiency *E_glob_*, local efficiency *E_loc_*, and assortativity *r*. In addition to those global metrics, we computed the small-world parameters [10], including clustering coefficient *C*, characteristic path length *L*, normalized clustering coefficient γ, normalized characteristic path length λ, and small-worldness scalar σ. Small-world network should satisfy the conditions of γ>1 and λ ≈ 1 (i.e., σ>1). Although we calculated all the above-mentioned global metrics, we reported mainly on *r*, *C*, *L*, and σ because some metrics bore a certain relationship (i.e., proportional or inverse) to other metrics. For example, clustering coefficient *C* bears a proportional relationship to local efficiency *E_loc_*, while characteristic path length *L* is inversely proportional to global efficiency *E_glob_* [62]. All metrics were calculated using the Brain Connectivity Toolbox (http://www.brain-connectivity-toolbox.net/).

While each of the metrics is highly dependent upon the threshold, there is no standard threshold currently accepted for the network construction. To avoid arbitrariness in network thresholding, we calculated the area under the curve (AUC) of each metric. The AUC provides an integrated scalar value representing the metric under investigation across the examined range of sparsity. The robustness of the AUC analysis for metrics has been demonstrated in previous studies [28], [63].

### 6. Hubness

Hubs are often identified using degree *k*, betweenness *b*, and nodal efficiency *e*. High-degree nodes can be considered as centers for information integration; those with high betweenness may serve as way stations for network traffic, and those with high nodal efficiency have superior information propagation ability and hence contribute to efficient information flow [13], [64]. In this study, hubs were defined as nodes with a *k*, *b*, or *e* value more than one standard deviation above the mean of all the nodes in the network [15].

### 7. Hub Disruption Index

Differences in local metrics are usually evaluated at each nodal level rather than at the network level. The hub disruption index is a useful metric for summarizing and visualizing the pattern of nodal abnormalities in a clinical group compared to a neurotypical group [65].

We calculated the hub disruption index, *κ*, in order to evaluate alteration in network topology in each individual brain, with reference to the normative network topology of the NC group. For each node in the network, we first subtracted the mean local metric (e.g., degree) in the NC group from the normalized degree in a participant, and then plotted the value against the mean of the NC group. We estimated slope using linear regression analysis, and defined the obtained slope as *κ*. When the resulting data are scattered along a horizontal line (*κ* = 0), there is no disruption in that participant. In contrast, data scattered along a negatively sloping line (*κ <*0) indicates some disruption in that participant. For instance, in comatose patients, high-degree nodes in the NC group showed a significant reduction of degree in the patients, while some low-degree nodes in the NC group showed a significant increase of degree in the patients, resulting in a significant negative slope for the patient group [65]. We expected that the ASC group would show some altered patterns in their local metrics. The hub disruption index was calculated separately for the three local metrics of degree *κ_D_*, betweenness *κ_B_*, and nodal efficiency *κ_E_*. Of note, the calculation of the hub disruption index was performed on the normalized AUC metric of each local metric.

### 8. Statistical Analysis

#### 8.1. Differences in network metrics

For between-group comparison of each of the metrics described above, permutation-based nonparametric tests with 5000 permutations were performed on the AUC of that metric, including age and gender as nuisance covariates. For each measure over the range of thresholds, two-sample two-tailed *t*-tests were performed in order to evaluate between-group differences, and a false discovery rate (FDR) correction was used for multiple comparisons [66].

#### 8.2. Reproducibility of hubs

As described previously, hubs were identified using a predefined threshold for degree *k*, betweenness *b*, or nodal efficiency *e*. Although several studies have identified functional hubs according to this manner, it is difficult to confirm whether or not those hubs are reproducible. In this study, we evaluated reproducibility using the bootstrapping method [67]. Within each group, each of the local metrics was resampled, and then hubs were identified using the resampled data and a predefined threshold. This procedure was repeated 10,000 times, and then we counted the occurrence frequency of hubness at each node. Finally, we computed the summation of the frequencies across all the three metrics at each node as a score of hubness of the node. We regarded a node as a functional hub if its score was higher than 2.1 (i.e., the frequency at each local metric was greater than 0.7 on average).

#### 8.3. Relationship between network metrics and AQ score

If a between-group difference in any of the global metrics or hub disruption indices was observed, we further investigated the relationship between the metric and the AQ score using a partial correlation analysis, with age and gender as controlling variables. For the local metrics, we repeated the partial correlation analysis only on nodes where significant group difference was found on all the three local metrics without correction for multiple comparisons, to minimize the problem of multiple comparisons.

## Results

### 1. Global Network Metrics

**Figure 1:**
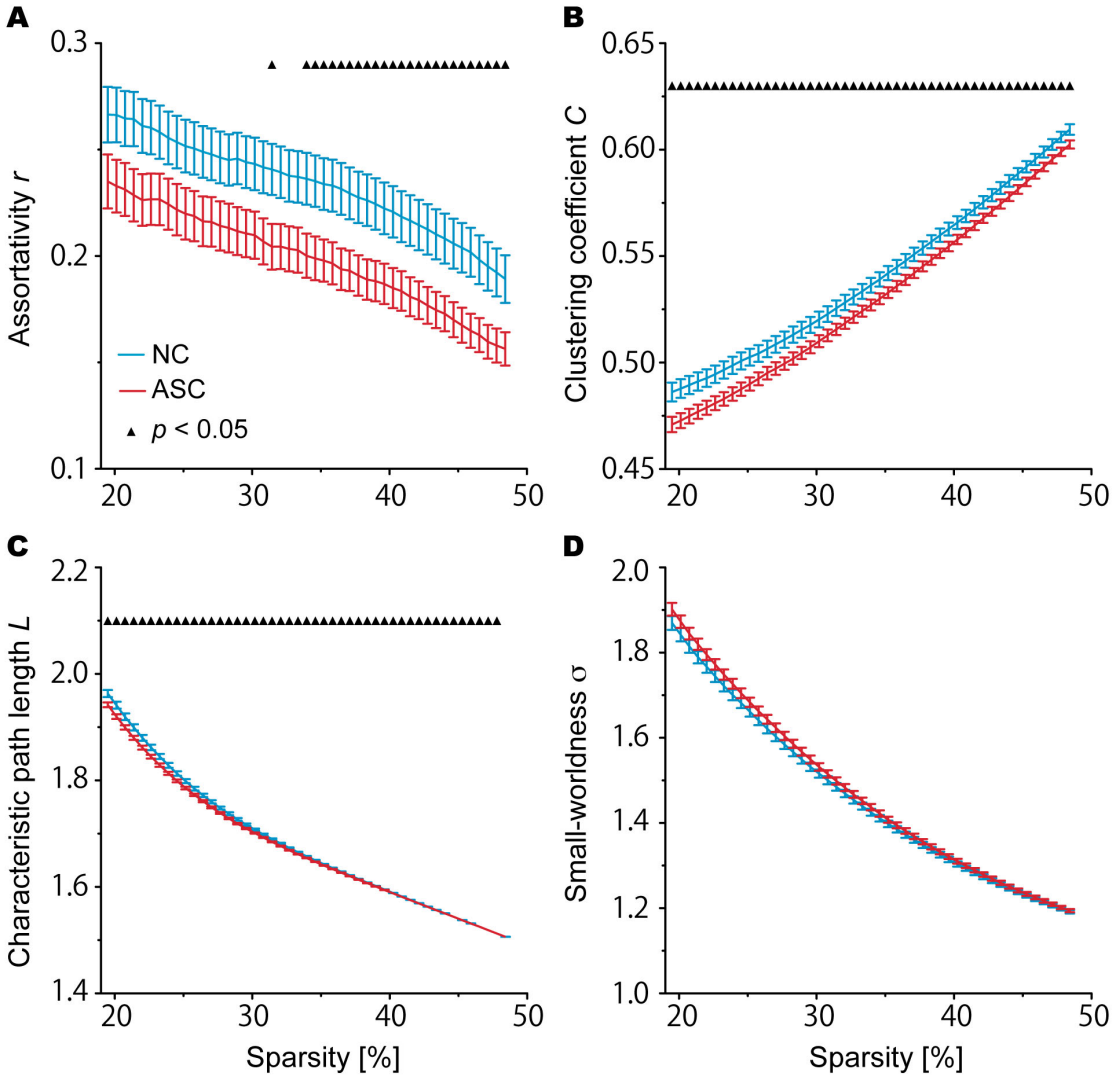
Global metrics of the assortativity, *r*; clustering coefficient, *C*; characteristic path length, *L*; and small-worldness scalar, σ as functions of the sparsity threshold. The ASC group (red line) exhibited lower *C* (B) and *L* (C) than the NC group (blue) over the range of sparsity thresholds (*p*<0.05, FDR corrected), while σ was comparable between groups (D). In addition, *r* was significantly lower in participants with ASC than in NCs over 33.9% of sparsity threshold values (A). The error bar indicates the standard error of the mean (SEM).

**Figure 2:**
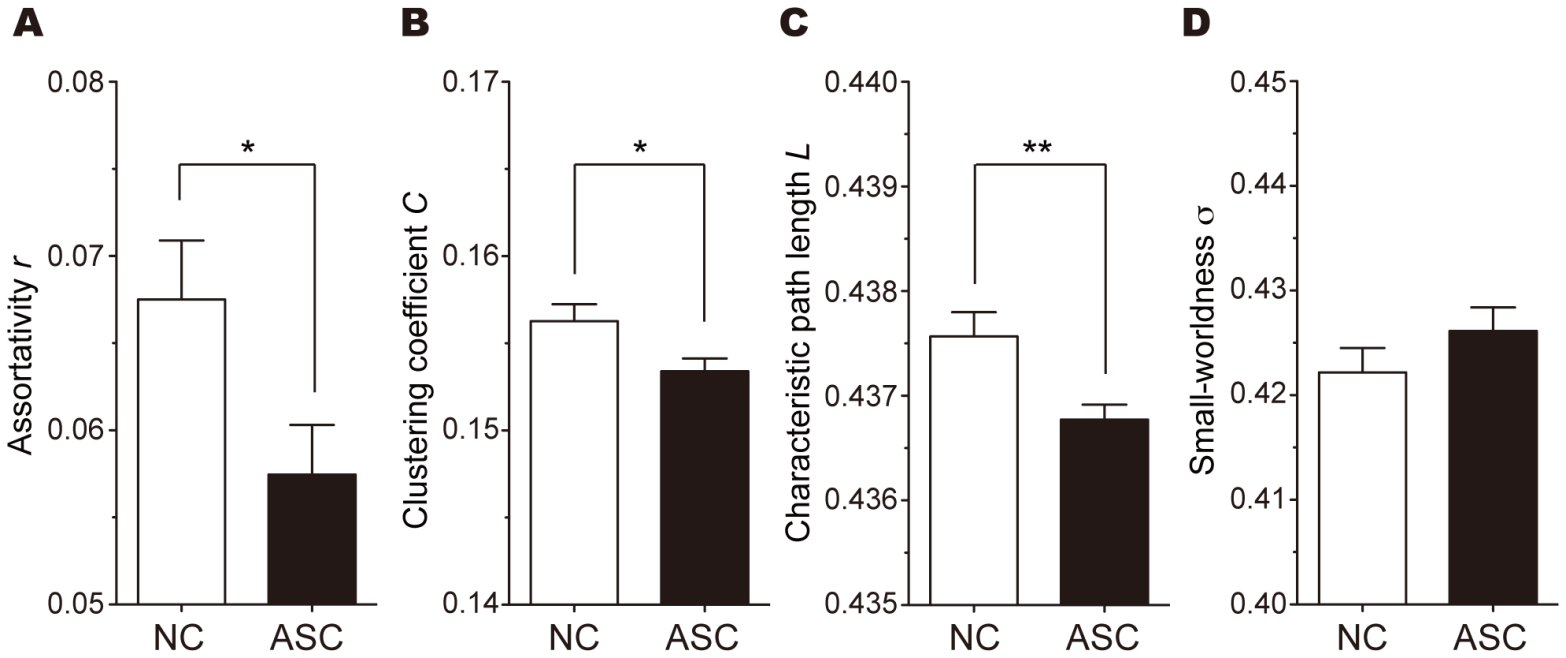
Between-group differences in the AUC values of assortativity, *r*; clustering coefficient, *C*; characteristic path length, *L*; and small-worldness scalar, σ. In the AUC analyses, participants in the ASC group (black) also showed significantly lower *r* (*p* = 0.013) (A), *C* (*p* = 0.014) (B), and *L* (*p* = 0.002) (C) than those in the NC group (white), whereas there were no significant differences in the small-worldness scalar σ (D) between the groups. The error bar indicates the standard error of the mean (SEM). Significance levels are represented by *p<0.05 and **p<0.01, respectively.

The global metrics of assortativity *r*, clustering coefficient *C*, characteristic path length *L*, and small-worldness σ in each group are depicted in Figure 1. Figures 1A to D represent the mean and standard error of these metrics, calculated at each sparsity level. For other global metrics (i.e., global efficiency *E_glob_*, local efficiency *E_loc_*, normalized clustering coefficient γ, and normalized characteristic path length λ), see Figures S2 and S3. As shown in Figure 1D and Figures S2C and S2D, the networks of both the NC and ASC groups exhibited the small-world properties (γ>1, λ ≈ 1, and σ>1) over the entire range of thresholds. The AUC analyses revealed that, while σ was comparable between the groups, *r*, *C*, and *L* were significantly lower in participants with ASC than in NCs (*r*: *p* = 0.013; *C*: *p* = 0.014; *L*: *p* = 0.002) (Figure 2).

### 2. Local Network Metrics

**Figure 3:**
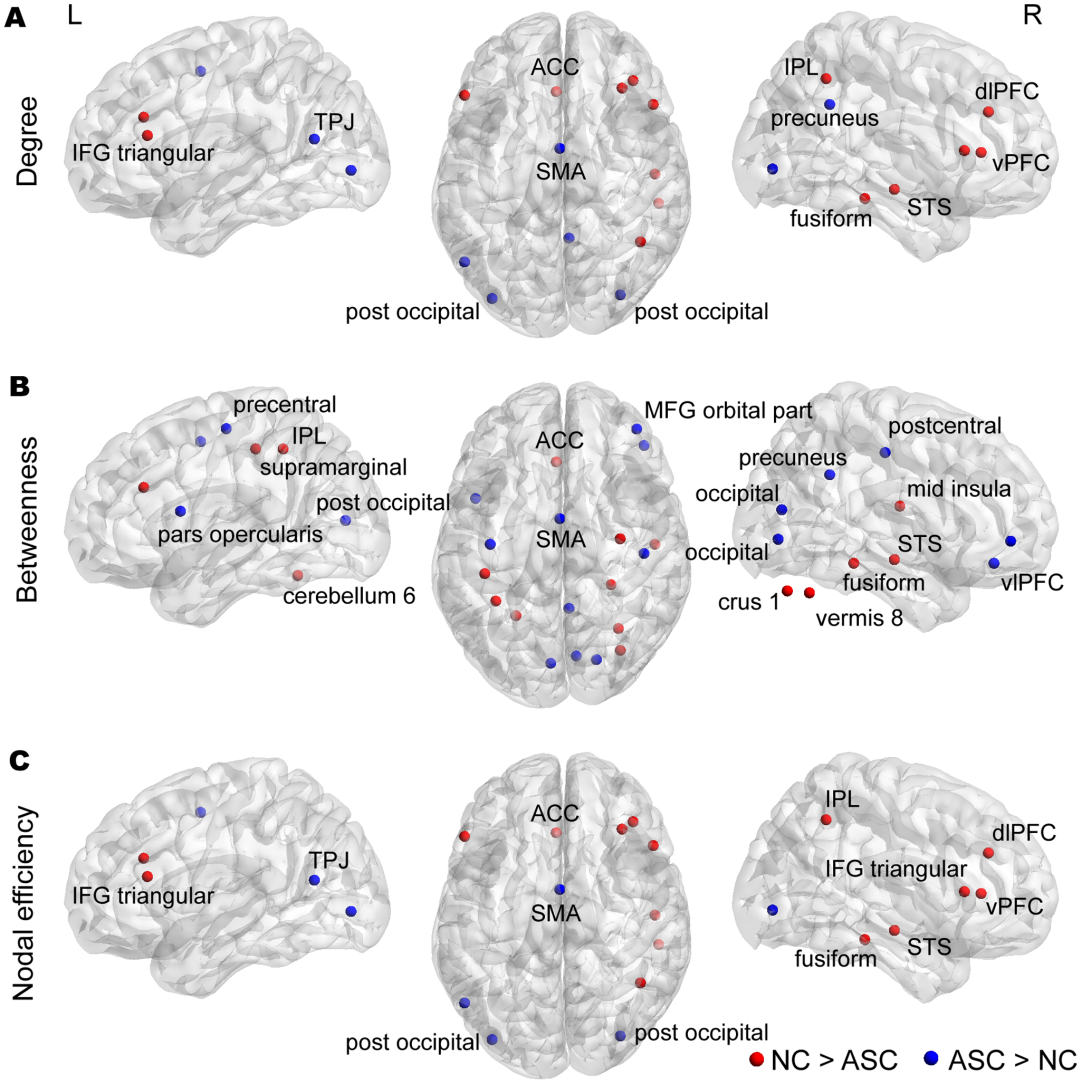
Altered local metrics of degree; betweenness; and nodal efficiency. Alterations in each of local metrics, including degree (A), betweenness (B), and nodal efficiency (C), were observed. The red sphere indicates NC>ASC, while the blue sphere denotes ASC>NC. Of note, in this study, the right inferior temporal [52, −15, −13] was regarded as the right superior temporal sulcus (STS), because this node was anatomically located on the STS rather than the inferior temporal. See Table S3 for other nodes re-labeled in this study. The distributions of nodes showing altered local metrics were visualized with the Brain Net Viewer (http://www.nitrc.org/projects/bnv/).

Group comparisons of the local metrics are shown in Figure 3 and Table S2. In total, we identified 28 nodes showing a significant group difference (*p*<0.05) in at least 1 metric: 5 in the fronto-parietal (FP) network, 6 in the cingulo-opercular (CO) network, 4 in the default-mode (DEF) network, 5 in the occipital (OC) network, 5 in the sensorimotor (SE) network, and 3 in the cerebellar (CER) network. While increased local metrics in the ASC group were mainly observed in the OC network, reductions were found in the FP and CO networks (Table 2). Notably, all three local metrics were significantly decreased at the left anterior cingulate cortex (ACC) [−2, 30, 27] and right superior temporal sulcus (STS) [52, −15, −13] and increased at the supplementary motor area (SMA) [0, −1, 52] in the ASC group.

**Table 2:** The number of affected nodes (NC>ASC or ASC>NC) and non-affected nodes.

Type†	Affected nodes††		Non-affected nodes††
	NC>ASC	ASC>NC	
FP (21)	4 (19%)	1 (4.8%)	16 (76.2%)
CO (32)	4 (12.5%)	2 (6.3%)	26 (81.3%)
DEF (34)	2 (5.9%)	2 (5.9%)	30 (88.2%)
OC (22)	0 (0%)	5 (22.7%)	17 (77.3%)
SE (33)	2 (6.1%)	3 (9.1%)	28 (84.9%)
CER (18)	3 (16.7%)	0 (0%)	15 (83.3%)

† The numbers in parentheses indicate the number of nodes in each sub-network.

†† The percentages in parentheses indicate the proportion of affected (or non-affected) nodes to the total number of nodes in each sub-network.

FP: fronto-parietal, CO: cingulo-opercular, DEF: default-mode, OC: occipital, SE: sensorimotor, CER: cerebellar.

### 3. Hubness

Hubs were identified using each of the normalized local metrics and the bootstrapping method described previously. For the sake of simplicity, a node is referred to as a *common* hub if that node was identified as a hub in both groups, and a node is referred to as a *group-specific* hub if that node was identified as a hub in only one group.

**Figure 4:**
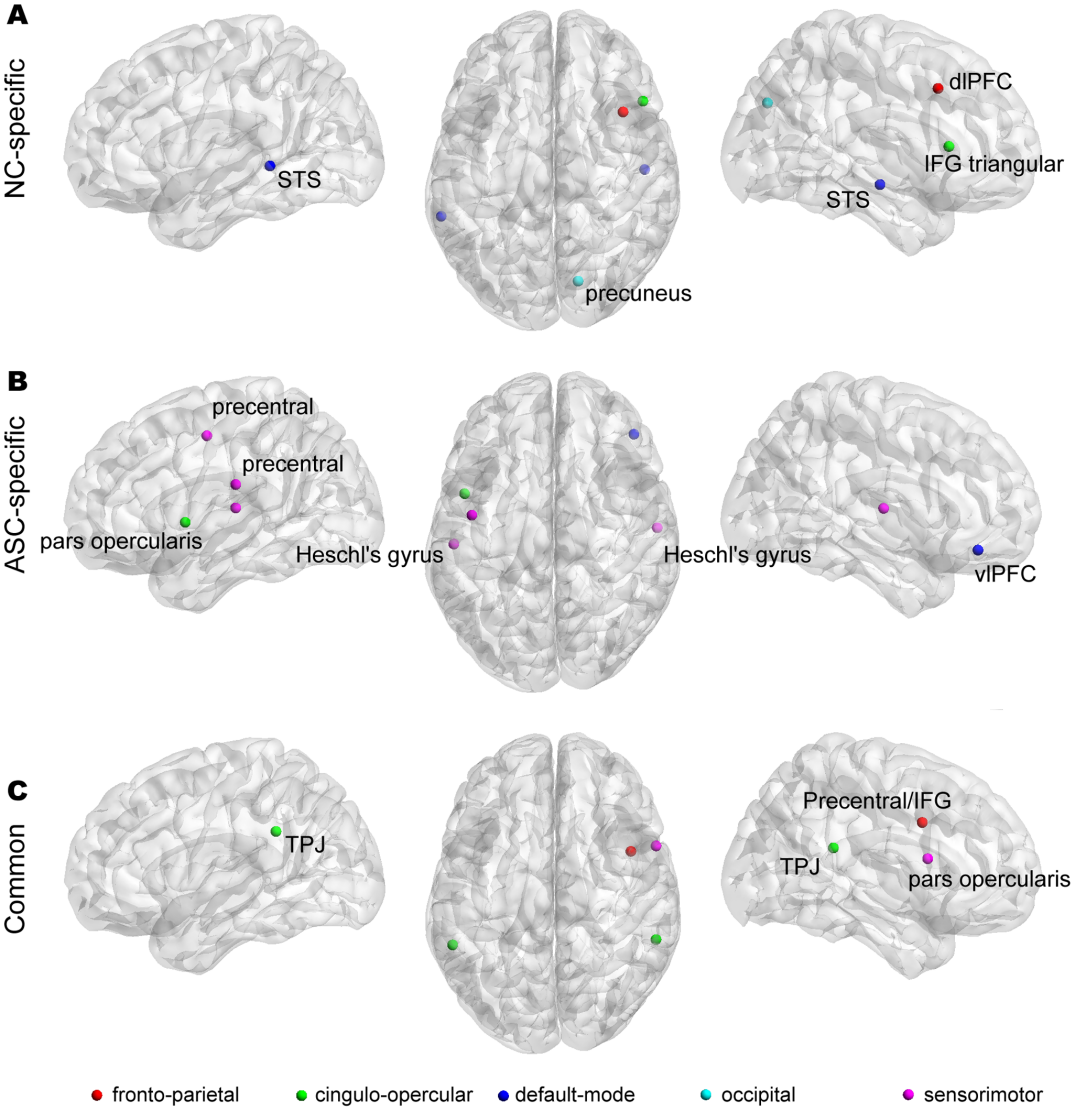
Hub nodes identified using the bootstrap method. The first row shows five NC-specific hubs (A), the second row shows six ASC-specific hubs (B), and the last row shows four common hubs (C). Hub distribution was visualized with the BrainNet Viewer (http://www.nitrc.org/projects/bnv/). Correspondences between colors and networks are as follows: fronto-parietal = red; cingulo-opercular = green; default mode = blue; occipital = cyan; sensorimotor = magenta.

In total, we identified 15 nodes that showed *common* or *group-specific* hub properties in the whole brain (Figure 4 and Table 3). Among those nodes, we found that five NC-specific hubs were in the right hemisphere (e.g., the bilateral STS, right dorsolateral prefrontal cortex (dlPFC), and precuneus), six ASC-specific hubs located bilaterally in the brain (e.g., the bilateral Heschl’s gyri and bilateral precentral gyri), and four common hubs located mainly in the frontal and parietal regions (the bilateral temporoparietal junction (TPJ), right inferior frontal gyrus (IFG) pars opercularis, and precentral/IFG). For functional hubs identified based on each local metric, see Figures S4, S5, and S6. The existence of group-specific hubs indicates an alteration of hub organization in the ASC group.

**Table 3:** A list of hub nodes identified using the Bootstrapping method.

Label	Coordinate	Type	Hub score†	
			NC	ASC
**NC-specific hubs**				
dlPFC R	40, 17, 40	FP	**2.48**	1.61
IFG triangular R	51, 23, 8	CO	**2.95**	1.10
STS R	52, −15, −13	DEF	**2.15**	0.38
STS L	−61, −41, −2	DEF	**2.43**	1.19
Precuneus R	15, −77, 32	OC	**2.31**	1.08
**ASC-specific hubs**				
IFG pars opercularis L	−48, 6, 1	CO	1.86	**2.85**
vlPFC R	46, 39, −15	DEF	0.45	**2.76**
Precentral L	−44, −6, 49	SE	1.75	**2.82**
Precentral L	−54, −22, 22	SE	1.12	**2.66**
Heschl’s gyrus R	59, −13, 8	SE	1.93	**2.69**
Heschl’s gyrus L	−54, −22, 9	SE	1.16	**2.32**
**Common hubs**				
Precentral/IFG R	44, 8, 34	FP	**2.71**	**2.28**
TPJ R	58, −41, 20	CO	**2.42**	**2.12**
TPJ L	−55, −44, 30	CO	**2.35**	**2.55**
IFG pars opercularis R	58, 11, 14	SE	**2.88**	**2.99**

† Hub score was calculated as the summation of frequencies across all the three local metrics, and then a node with high hub score (≥2.1) was considered as a hub in this study.

FP: fronto-parietal, CO: cingulo-opercular, DEF: default-mode, OC: occipital, SE: sensorimotor.

### 4. Hub Disruption Indices

**Figure 5:**
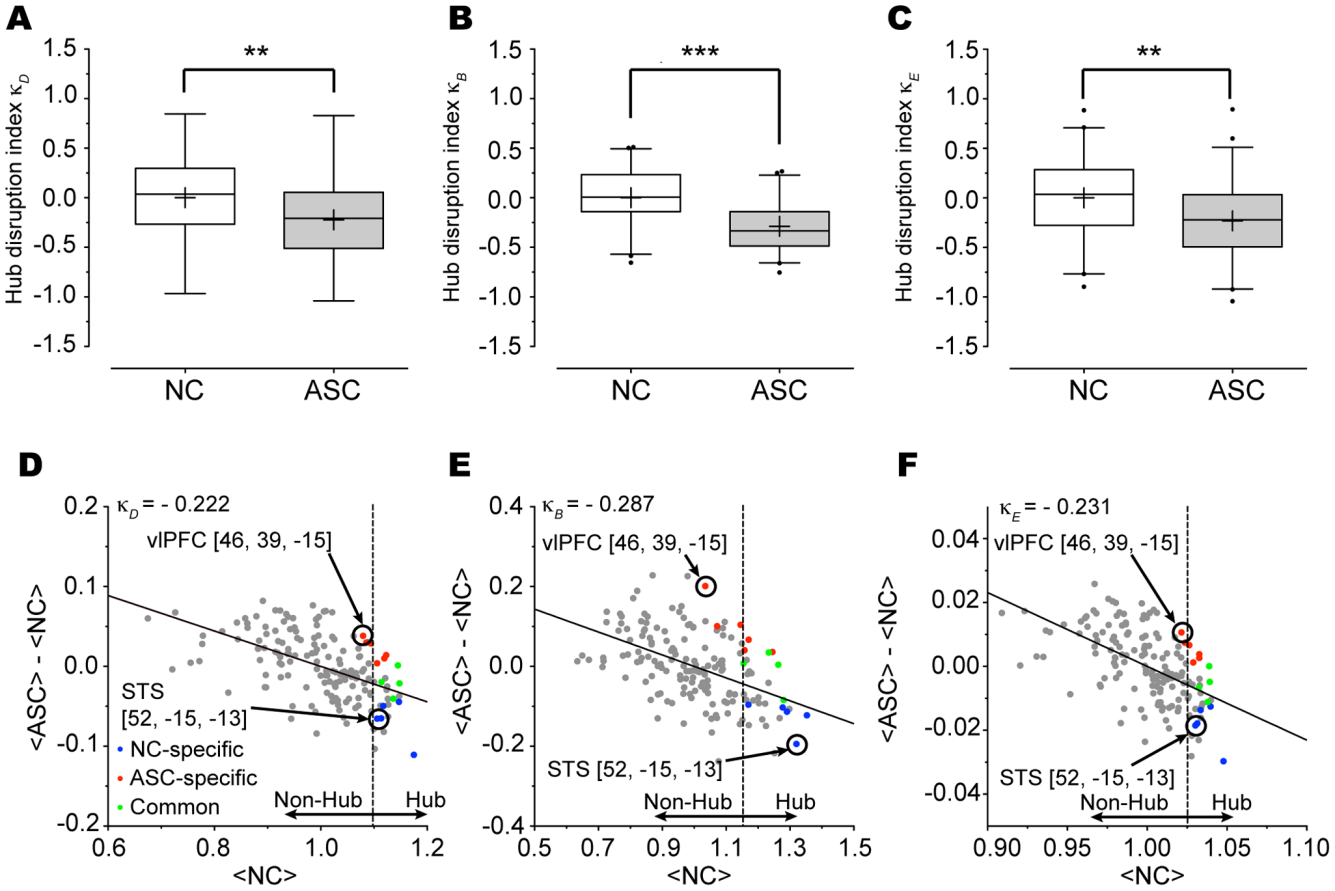
Hub disruption index for each of the three normalized local network metrics of degree; betweenness; and nodal efficiency. The ASC group (gray) exhibited significant reductions in all the three hub disruption indices (*κ_D_*: *p* = 0.007; *κ_B_*: *p*<0.001; *κ_E_*: *p* = 0.006) (A, B, and C). For illustrative purposes, each of the local metrics was averaged, and then the hub disruption index was calculated at the group level (D, E, and F). Each of hub disruption indices showed significantly negative values in the ASC group, indicating the disruption of hub organization. The colors represent the following groups: NC-specific hub: blue, ASC-specific hub: red, common hub: green. Of note, nodes with high hub scores (≥2.1) were considered as hubs in this study. Significance levels are denoted by **p<0.01 and ***p<0.001, respectively.

Consistent with the possibility of altered hub organization in ASC, the hub disruption index *κ* exhibited significant negative values for the ASC group in all three metrics (*κ_D_*: *p* = 0.007; *κ_B_*: *p*<0.001; *κ_E_*: *p* = 0.006) (Figures 5A to C). For instance, the right STS consistently held hub properties across all three local metrics in the NC group, and its averaged network topology in the ASC group was smaller than the normative network topology of the NC group; on the other hand, the right ventrolateral PFC (vlPFC) [46, 39, −15] consistently held hub properties across all three local metrics in the ASC group, and its averaged network topology in the ASC group was greater than the reference calculated from the NC group (Figures 5D to F).

### 5. Relationship between Altered Network Topology and the AQ Score

**Figure 6:**
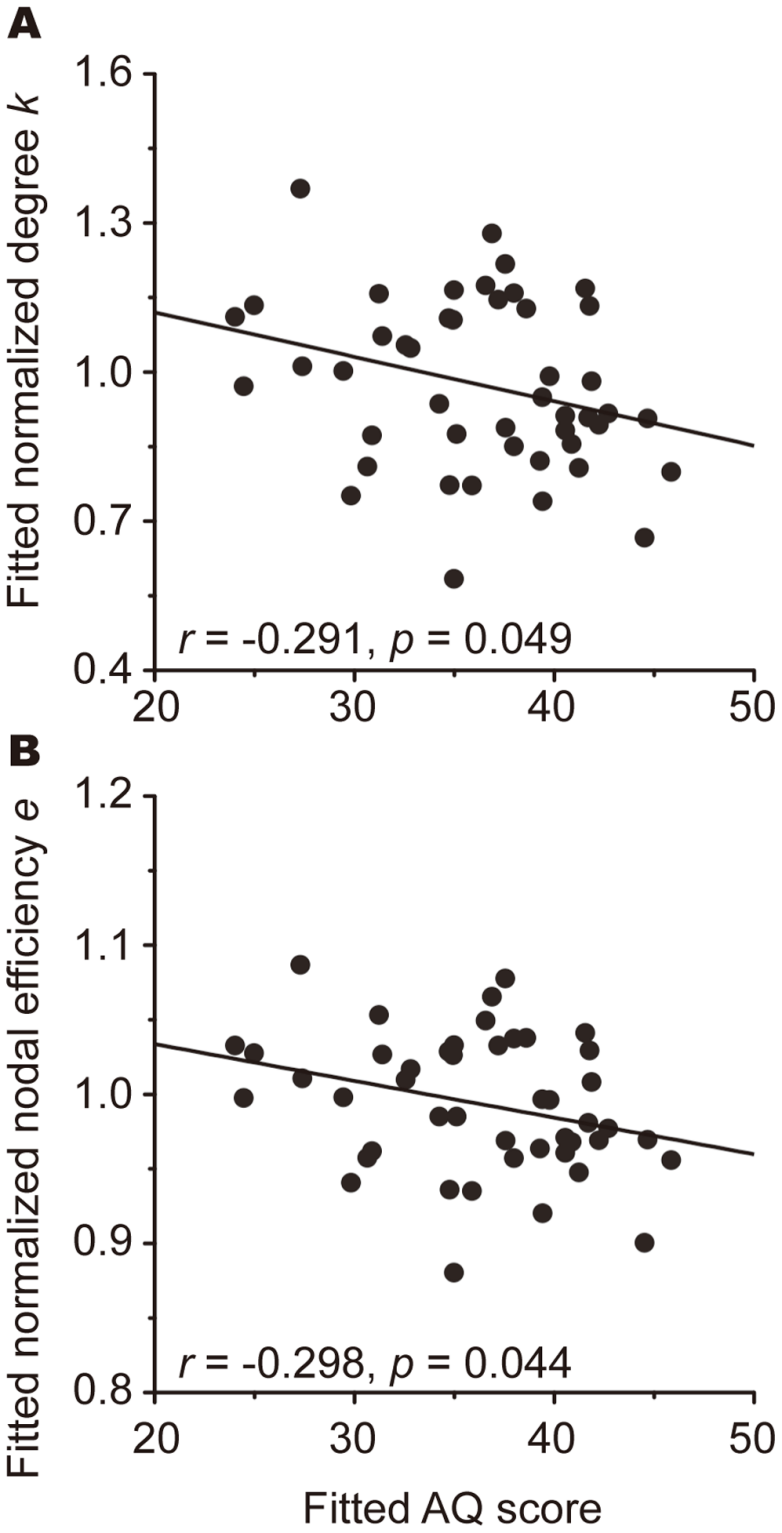
Scatter plots of degree and nodal efficiency of the left ACC against AQ score. Partial correlation analyses revealed that there were the significant negative correlations between the AQ score and two of the local metrics of the ACC (*k*: *r* = −0.291, *p* = 0.049; *e*: *r* = −0.298, *p* = 0.044).

No significant correlation was found between the AQ score and any of the global metrics or any of the hub disruption indices. For the local metrics, we only focused on nodes that showed significant alterations in all three local metrics. Thus, the left ACC, SMA, and right STS were selected as candidates for further partial correlation analyses. As shown in Figure 6, we found significant correlations between the AQ score and two of the local metrics of the ACC (*k*: *r* = −0.291, *p* = 0.049; *e*: *r* = −0.298, *p* = 0.044), suggesting that the severity of autistic traits may be reflected in the alterations of nodal metrics.

## Discussion

This study examined the topological properties of the resting-state functional brain network in participants with ASC using a graph-theoretical analysis. Participants with ASC showed significant decreases in clustering coefficient *C* and characteristic path length *L*, which is consistent with previous findings in adolescents with ASC [39]. Furthermore, reduced assortativity was found in ASC, implying that high-degree nodes tended to connect with low-degree nodes to a greater degree in the ASC brain than in the neurotypical brain. These findings are consistent with the view that network organization in the ASC brain shifts toward randomization compared to that in the NC brain [39], [68]. In analyses of the local metrics, increases were observed primarily in the occipital (OC) and sensorimotor (SE) networks, while reductions were found in the fronto-parietal (FP) and cingulo-opercular (CO) networks in participants with ASC, compared with NCs. We also observed that, while both groups shared some nodes as common hubs, each group had several group-specific hubs, indicating changes in hub organization in ASC. Indeed, such changes were confirmed by analyses of all the three hub disruption indices. Subsequent partial correlation analyses demonstrated significant associations between the total AQ score and two local metrics (degree *k* and nodal efficiency *e*) of the left ACC. These findings indicate that the influence of nodes is significantly altered in the ASC brain and such alteration is particularly characterized by changes of “hubness” in several nodes critical for social and non-social cognition, both of which are profoundly impaired in people with ASC.

### 1. Altered Global Network Organization in ASC

A recent study demonstrated that adolescents with ASC exhibited enhanced global integration (decreased characteristic path length *L*) and disrupted local segregation (decreased clustering coefficient *C*) compared with neurotypical counterparts [39]. Although almost all of the examined global metrics in adults with ASC were consistent with those previously reported for adolescents with ASC [39], only normalized clustering coefficient γ showed a discrepancy between adolescents and adults with ASC; while adolescents with ASC showed a significant reduction in this metric, it was comparable in adults (see Figure S2C). This discrepancy may reflect a normalization process from puberty to adulthood in ASC, which has been often observed for other structural measures (e.g., fractional anisotropy [69]). To investigate this possibility, future investigations need to directly compare data from the two developmental stages obtained in the same experimental setting.

In addition to *C* and *L*, we examined the assortativity *r* of the network; *r* quantifies whether nodes preferentially connect to nodes with similar degrees. A network is assortative if *r*>0 and random if *r* = 0 [70]. Compared with neurotypical brain networks, autistic brain networks were less assortative but not disassortative (i.e., *r*>0), providing further evidence of increased randomness in the network [39], [68]. Given that functional connectivity used for constructing a network in this study could be considered as an indirect measure of anatomical connectivity, disruptions of anatomical brain network may possibly induce atypical organization of functional brain network in ASC. Indeed, several diffusion tensor imaging (DTI) studies have demonstrated that people with ASC show alterations in local anatomical pathways, such as the inferior longitudinal fasciculus and uncinate fasciculus [71]. Although no DTI data are available to confirm the link between anatomical and functional brain networks of ASC on a topological space, our findings on global metrics indicate that the tendency of increased randomness in functional network may reflect disrupted anatomical network organization in the ASC brain.

### 2. Alterations of Local Metrics in ASC

At the nodal level, increased local metrics of degree *k*, betweenness *b*, and nodal efficiency *e*, were primarily observed within the occipital (OC) and sensorimotor (SE) networks, while reductions were mainly observed in the fronto-parietal (FP) and cingulo-opercular (CO) networks in ASC. The FP and CO networks are thought to be critical for adaptive control and stable set maintenance, respectively [72], [73]. Previous fMRI studies have revealed that individuals with ASC often show abnormal activation or atypical connections within the FP network during cognitive control and executive function tasks [74]–[76]. Furthermore, a recent fMRI study using pattern classification analysis demonstrated that the degree of nodes within the CO network contributed to improved classification of the diagnostic status between neurotypical and ASC individuals [36], indicating significant functional alterations in the CO network that may be characteristic to ASC.

Within the CO network, the left ACC exhibited significant reductions in all three local metrics, together with significant negative correlations between the AQ score and *k* and *e*. Previous studies on ASC have reported reduced brain activation in the ACC during a number of different cognitive tasks [77], [78], as well as during rest [3]. This region is thought to be important for various cognitive and affective functions, including emotional processing [79], conflict monitoring [80], and the learning and selection of high-level behavioral plans [81]. Together with these previous findings, our results suggest that malfunction of the ACC may contribute to impairments in these diverse cognitive and affective functions in ASC.

Notably, significant increases in all three local metrics were observed in the SMA, which is implicated in multiple roles mainly related to movement (e.g., memory-guided movement sequencing) [82]. Although motor dysfunctions may not be always included in the core clinical symptoms of ASC [83], [84], clinical observations related to abnormal motions are frequently reported in people with ASC, raising the possibility that people with ASC may have motor network dysfunctions. Indeed, several neuroimaging studies have reported that people with ASC exhibit altered brain activity or functional connectivity in the SMA during motor tasks [85]–[88]. For example, Takarae et al. demonstrated reduced brain activation in the portion of the SMA (supplementary eye field) during visually guided saccades [87]. Taken together, increased local metrics in this node may reflect such atypical brain activation or connectivity that underlies motor dysfunctions in ASC.

### 3. Loss of Hubs in the Social-Communication Network of Autism

Several studies have identified functional hubs using several topological metrics (e.g., degree, betweenness, and nodal efficiency) [13]–[17], [89]. In this study, most of the previously reported hubs were replicated using at least one of the three local metrics (e.g., the right precuneus, anterior insula, left inferior parietal lobule) (see Figures S4, S5, and S6), except in a few nodes (e.g., the SMA). Notably, four nodes (the right precentral/IFG, bilateral TPJ, and right IFG pars opercularis) were identified as common hubs, while five nodes (the bilateral STS, right dlPFC, and right precuneus) and six nodes (the right vlPFC, left IFG pars opercularis, bilateral precentral gyri, and bilateral Heschl’s gyri) were identified as NC-specific and ASC-specific hubs, respectively.

Several lines of evidence suggest that dysfunction of the mirror neuron system (MNS) may account for a socio-communicative deficit, which is one of the core clinical symptoms in people with ASC [90], [91]. This system involves the right IFG and STS [92] and is thought to represent the actions of others and intentions associated with those actions. The right IFG is one of the central nodes in the MNS and is involved in directing social interaction [93]. Several studies have reported morphological and functional abnormalities in ASC, including reduced gray matter volume [94], [95] and reduced neural activity during social tasks [96]. In this study, the right IFG pars opercularis was determined to be a hub in both NC and ASC networks, whereas functional connections between this node and several nodes (e.g., the right anterior insula and left ACC) were decreased for ASC, as indicated by functional connectivity analysis (see Figure S7 and Table S4). Although no over-connections with this node were observed in this study, a recent fMRI study reported over-connectivity between this region and others, involving the bilateral OFC, right putamen, and accumbens [97]. Therefore, the observation that the right IFG retained hubness in ASC in the presence of several reduced functional connections may be suggestive of the existence of complementary over-connection with other nodes, possibly at sub-threshold levels.

The right STS was the only NC-specific hub and showed significant reductions across all three local metrics in the ASC group. This region has been demonstrated to play key roles in several social perceptions, such as the perception of eye gaze and biological motion [93]. This node, particularly the posterior part of the STS, has been proposed as one of the core regions responsible for social perceptual impairments in ASC [98]. Consistent with this view, a number of previous studies on ASC have reported abnormalities in this area, such as cortical thinning [99], volumetric reductions [100], under-connectivity between this node and other regions (e.g., amygdala) [101], and atypical functional segregation of the posterior STS [102]. In this study, no under-connections with this node were observed, and no significant associations between any of the local metrics of this node and the AQ score were found. However, in support of the view of mirror neuron dysfunction, the loss of hubness in the right STS together with significant reductions in all three local metrics may underlie socio-cognitive deficits in ASC.

It is noteworthy that six ASC-specific hubs, including the right vlPFC, bilateral precentral gyri, bilateral primary auditory cortices (Heschl’s gyri), and left pars opercularis, were identified in this study. As shown in some behavioral studies [103], [104], people with ASC have difficulties in the inhibition of inappropriate behaviors. A recent fMRI study demonstrated that, while neurotypical adults showed no recruitment of the right vlPFC, adults with ASC showed atypical hyper-recruitment of the right vlPFC during inhibition control to socially relevant stimuli [74]. Therefore, the hubness in this node may reflect the atypical functional circuitry in the ASC brain. In addition to deficits in social and communicative functions, people with ASC often show impairments in sensory processing. In particular, auditory hypersensitivity is one of the common sensory impairments in ASC, and several behavioral studies have reported deficits in auditory filtering [105]–[107]. A recent magnetoencephalographic study reported that children with ASC showed longer M50 and M100 peak latencies compared to neurotypical children and that those long latencies were negatively correlated with the severity of auditory hypersensitivity [108]. Furthermore, Hyde et al. recently reported increased cortical thickness in the bilateral primary auditory cortices in the ASC brain [109]. Although no clinical and behavioral data regarding altered sensations are available in this study, our findings on the primary auditory cortex may reflect abnormalities in auditory processing in ASC.

The altered hub organization revealed in this study was also summarized as a disruption of hub rank order. Even though declines in the hub disruption indices were moderate compared to those in comatose patients (ASC: *κ_D_* = −0.22; comatose [65]: *κ_D_* = −0.82), the ASC group still showed clear significant reductions of the indices in all three local metrics such that some hubs in the normal brain network (e.g., the right STS) were non-hubs in the ASC brain. Although no significant correlations between hub disruption indices and the AQ score were found in this study, future studies are needed to address the possibility that a range of dysfunctions involving socio-communicative deficits, dyspraxia, auditory hypersensitivity in ASC might arise from changes in the order of node importance in functional brain network.

Consistent with our results regarding hubness and hub disruption indices, functional connectivity analyses revealed weakened functional connections between nodes in the large-scale system composed of the MNS, limbic system, and insula (see Text S2 and Table S4). The insula is anatomically connected with brain regions within the MNS and limbic system, and this system as a whole has been proposed to facilitate the understanding of other people’s emotions through action representation [110]. Therefore, our results suggest that poor communication between the MNS and limbic system through the anterior insula may contribute to social cognitive and affective dysfunctions in ASC. In addition, reduced functional connections were also found in the motor network involving the precentral gyrus, cerebellum, and putamen, which may potentially explain ASC-associated motor deficits [86], [87], [111], [112]. Taken together, our findings on functional connectivity are in line with the view that ASC is characterized by an array of dysfunctions including the hallmark deficits in social and communicative functions.

It would also be important in future studies to link the observed pattern of altered functional networks with molecular and genetic findings to advance our understanding of the pathological mechanisms of ASC. Molecular basis of ASC is associated with many of the synaptic cell-adhesion molecules, including neurexins, neuroligins, and cadherins [113]–[116], all of which play important roles in determining aspects of neural connections including synaptic formation and axonal guidance. Altered expressions of genes encoding these molecules might lead to the development of altered functional network for ASC that displays the characteristics of brain connectivity revealed by the present study.

### 4. Limitations

There are several limitations in the present study. Firstly, functional brain networks were constructed using the 160 functional ROIs defined by Dosenbach et al. [55], while previous studies have often employed anatomical ROIs (e.g., an automated anatomical labeling atlas). There is evidence that differences in node definition can affect the resulting topological architecture [117]–[119]. In addition, the set of nodes used in this study does not include certain limbic regions (e.g., amygdala) that are important in clinical populations. However, the set of nodes used here exhibited better reliability than the anatomical scheme for calculating the network topology [120], and the same node definition was used in several network studies [17], [36]. Moreover, the fact that our findings on global metrics were consistent with the results of a previous study of adolescents with ASC [39] further supports the validity of our analyses.

Secondly, the frequency band of the analyzed resting-state brain activity was restricted to the low frequency range due to limitations in the temporal resolution of fMRI, and the relatively sluggish nature of the hemodynamic response. In this study, we applied a band-pass filter (0.009–0.08 Hz) to remove artifacts, because low-frequency spontaneous fluctuations of rs-fMRI signals within this range are thought to reflect neurophysiological processes of the human brain [121], [122]. On the other hand, abnormal brain activities in different frequency bands have been reported in people with ASC based on electroencephalography (EEG) data [31], [34]. In future studies, it will be important to examine the topological properties of the functional brain network in individuals with ASC using both electrophysiologically and hemodynamically derived signals.

Thirdly, some demographic information (IQ and handedness) was missing in this study. To validate our main findings, we repeated the second-level analyses of all the 8 global metrics on 39 NCs and 45 participants with ASC, and confirmed that, in spite of reduced statistical power, statistical conclusions were preserved for all the 8 measures (see Figure S8). Although gender differences might be one of the confounding factors, the small number of female participants (*n* = 7 in each group) did not allow us to draw statistically rigorous conclusions regarding possible gender effects on the network measures. Further investigations will be needed to examine the gender effects on the topological metrics.

Lastly, we examined the topological properties of the resting-state functional brain network, while state-dependent topological changes have been reported in ASC [36] and schizophrenia [25]. Hence, exploring network topologies during tasks would also be important for developing a complete understanding of the ASC brain.

## Conclusions

Our findings revealed significant alterations in global network topologies, as well as changes in the order of node importance and the loss of hubness associated with social and non-social functions in ASC. Consistent with previous findings [39], [68], our results suggest that the autistic brain network is likely to be randomized compared to the neurotypical one. At the nodal level, the left ACC and right STS might contribute to socio-communicative deficiencies in ASC. Advances in the modeling of functional brain networks have provided powerful methodological frameworks to reveal the neuronal alterations underlying atypical behaviors in ASC.

## Supporting Information

Figure S1 **Effects of the scrubbing method.** Using the frame-wise displacement metric with a 0.5 mm threshold, the motion-contaminated volumes were detected in 16 NCs and 12 participants with ASC, respectively. The first row shows the effects of the scrubbing method for the average of 16 NCs (A to C), and the second row shows the effects for the average of 12 participants with ASC (D to F). The first column shows the correlation matrices for both groups, calculated without applying the scrubbing method (for simplicity, we will refer these as “standard”) (A and D); the second column shows the correlation matrices calculated with the scrubbing method (henceforth, we will refer these as “scrubbed”) (B and E); the last column shows the difference between the standard and scrubbed matrices (C and F). We observed slightly decreased correlation values in the short-range, and increased correlation values in the long-range after adopting the scrubbing method. For example, the correlation between the right vPFC [34], [32], [7] and right vlPFC [39], [42], [16] was decreased after the removal of motion-contaminated volumes (*D* = 14.25 mm; *Δr* = −0.01), while the correlation between the right vmPFC [6], [64], [3] and left post occipital [−37, −83, −2] was increased after scrubbing (*D* = 153.24 mm; *Δr* = 0.016). FP: fronto-parietal, CO: cingulo-opercular, DEF: default mode, OC: occipital, SE: sensorimotor, CER: cerebellar, *D*: the Euclidean distance between node A and node B.
(TIF)

Figure S2 **Global metrics of global efficiency,** ***E_glob_*** **, local efficiency,** ***E_loc_*** **, normalized clustering coefficient, γ, and normalized characteristic path length, λ, as functions of the sparsity threshold.** The error bar indicates the standard error of the mean (SEM). Compared with the NC group (blue line), the ASC group (red line) showed significantly higher *E_glob_* and lower *E_loc_* and λ (*p*<0.05, FDR corrected) over the range of sparsity thresholds (A, B, and D), whereas γ was comparable between the groups (C).
(TIF)

Figure S3 **Between-group differences in the AUC values of global efficiency,** ***E_glob_*** **, local efficiency,** ***E_loc_*** **, normalized clustering coefficient, γ, and normalized characteristic path length, λ.** In the AUC analyses, participants with ASC (black) exhibited significantly higher *E_glob_* (*p* = 0.02) (A), and significantly lower *E_loc_* (*p* = 0.011) (B) and λ (*p* = 0.001) (D), while γ was comparable between the groups (D). Significance levels are represented by *p<0.05 and **p<0.01, respectively.
(TIF)

Figure S4 **Functional hubs were identified using degree and bootstrapping method.** The first row shows ten “NC-specific” hubs, involving the bilateral anterior insula, left STS, the bilateral dlPFC, and right IFG triangular; the second row shows six “ASC-specific” hubs, including the right vlPFC, left precentral gyrus, and left Heschl’s gyrus; and the last row shows five “common” hubs (the right dlPFC, left IPL, TPJ, and left IFG pars opercularis). Correspondences between colors and networks are as follows: fronto-parietal = red; cingulo-opercular = green; default mode = blue; sensorimotor = magenta. Hubs were visualized with the BrainNet Viewer (http://www.nitrc.org/projects/bnv/).
(TIF)

Figure S5 **Functional hubs were identified using betweenness and bootstrapping method.** The first row shows eight “NC-specific” hubs, involving the bilateral STS, left precuneus, the bilateral crus 1, and right IFG triangular; the second row shows 11 “ASC-specific” hubs, including the SMA, the right vlPFC, left TPJ, and right Heschl’s gyrus; and the last row shows seven “common” hub, encompassing the bilateral precuneus, the right TPJ, the right IFG pars opercularis, and left precentral. Correspondences between colors and networks are as follows: fronto-parietal = red; cingulo-opercular = green; default mode = blue; occipital = cyan; sensorimotor = magenta; cerebellar = yellow. Hubs were visualized with the BrainNet Viewer (http://www.nitrc.org/projects/bnv/).
(TIF)

Figure S6 **Functional hubs were identified using nodal efficiency and bootstrapping method.** The first row shows nine “NC-specific” hubs, involving the bilateral anterior insula, the left STS, the left ACC, the right dlPFC, and right IFG triangular; the second row shows five “ASC-specific” hubs, including the right vlPFC, left precentral, left angular gyrus, and left Heschl’s gyrus; and the last row shows seven “common” hub, encompassing the bilateral IFG pars opercularis, and right dlPFC. Correspondences between colors and networks are as follows: fronto-parietal = red; cingulo-opercular = green; default mode = blue; sensorimotor = magenta. Hubs were visualized with the BrainNet Viewer (http://www.nitrc.org/projects/bnv/).
(TIF)

Figure S7 **Reduced functional connectivity in participants with ASC identified using the network-based statistic approach.** The reduced connections were found mainly within the cingulo-opercular network rather than within the default mode network. Correspondences between colors and networks are as follows: fronto-parietal = red; cingulo-opercular = green; default mode = blue; sensorimotor = magenta; cerebellar = yellow. These reduced connections were visualized with the BrainNet Viewer (http://www.nitrc.org/projects/bnv/).
(TIF)

Figure S8 **Between group differences in the AUC values of all the global measures performed on 39 NCs and 45 participants with ASC.** The second-level analyses were re-conducted on 39 NCs and 45 ASC participants to ensure that between-group differences were not due to differences in handedness. Statistical conclusions were preserved for all the global metrics (clustering coefficient: *p* = 0.048, characteristic path length: *p* = 0.012; assortativity: *p* = 0.032; small-worldness: *p* = 0.083; global efficiency: *p* = 0.018; local efficiency: *p* = 0.044; normalized clustering coefficient: *p* = 0.170; normalized characteristic path length: *p* = 0.013). Significance levels are represented by *p<0.05 and **p<0.01, respectively.
(TIF)

Table S1 **Brief description of network metrics used in this study.**
(DOC)

Table S2 **Altered local network metrics (degree** ***k*** **, betweenness** ***b*** **, and nodal efficiency** ***e*** **) in the ASC group compared to the NC group.**
(DOC)

Table S3 **A list of re-labeled nodes in this study.**
(DOC)

Table S4 **Reduced functional connectivity in participants with ASC compared to NCs.**
(DOC)

Text S1 **The details of scrubbing method used in this study.**
(DOC)

Text S2 **Functional connectivity analysis.**
(DOC)
