# Supplementary figures and images for: Altered Network Topologies and Hub Organization in Adults with Autism: A Resting-State fMRI Study
Source: PLoS One. 2014 Apr 8;9(4):e94115. doi: 10.1371/journal.pone.0094115 (PMC3979738; doi:10.1371/journal.pone.0094115)

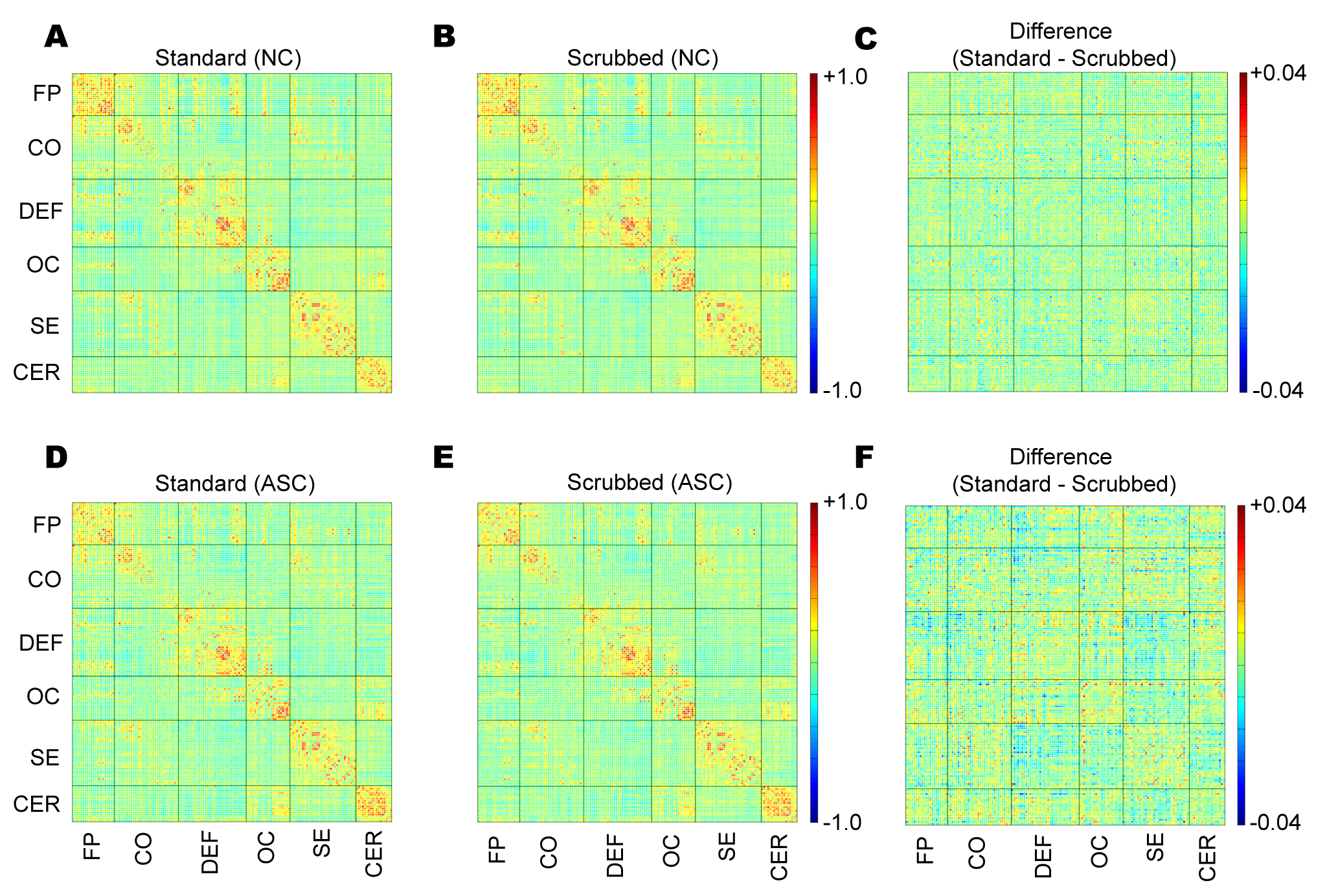

Supplement: Figure S1 — Effects of the scrubbing method. Using the frame-wise displacement metric with a 0.5 mm threshold, the motion-contaminated volumes were detected in 16 NCs and 12 participants with ASC, respectively. The first row shows the effects of the scrubbing method for the average of 16 NCs (A to C), and the second row shows the effects for the average of 12 participants with ASC (D to F). The first column shows the correlation matrices for both groups, calculated without applying the scrubbing method (for simplicity, we will refer these as “standard”) (A and D); the second column shows the correlation matrices calculated with the scrubbing method (henceforth, we will refer these as “scrubbed”) (B and E); the last column shows the difference between the standard and scrubbed matrices (C and F). We observed slightly decreased correlation values in the short-range, and increased correlation values in the long-range after adopting the scrubbing method. For example, the correlation between the right vPFC [34], [32], [7] and right vlPFC [39], [42], [16] was decreased after the removal of motion-contaminated volumes (D = 14.25 mm; Δr = −0.01), while the correlation between the right vmPFC [6], [64], [3] and left post occipital [−37, −83, −2] was increased after scrubbing (D = 153.24 mm; Δr = 0.016). FP: fronto-parietal, CO: cingulo-opercular, DEF: default mode, OC: occipital, SE: sensorimotor, CER: cerebellar, D: the Euclidean distance between node A and node B. (TIF) [file pone.0094115.s001.tif]

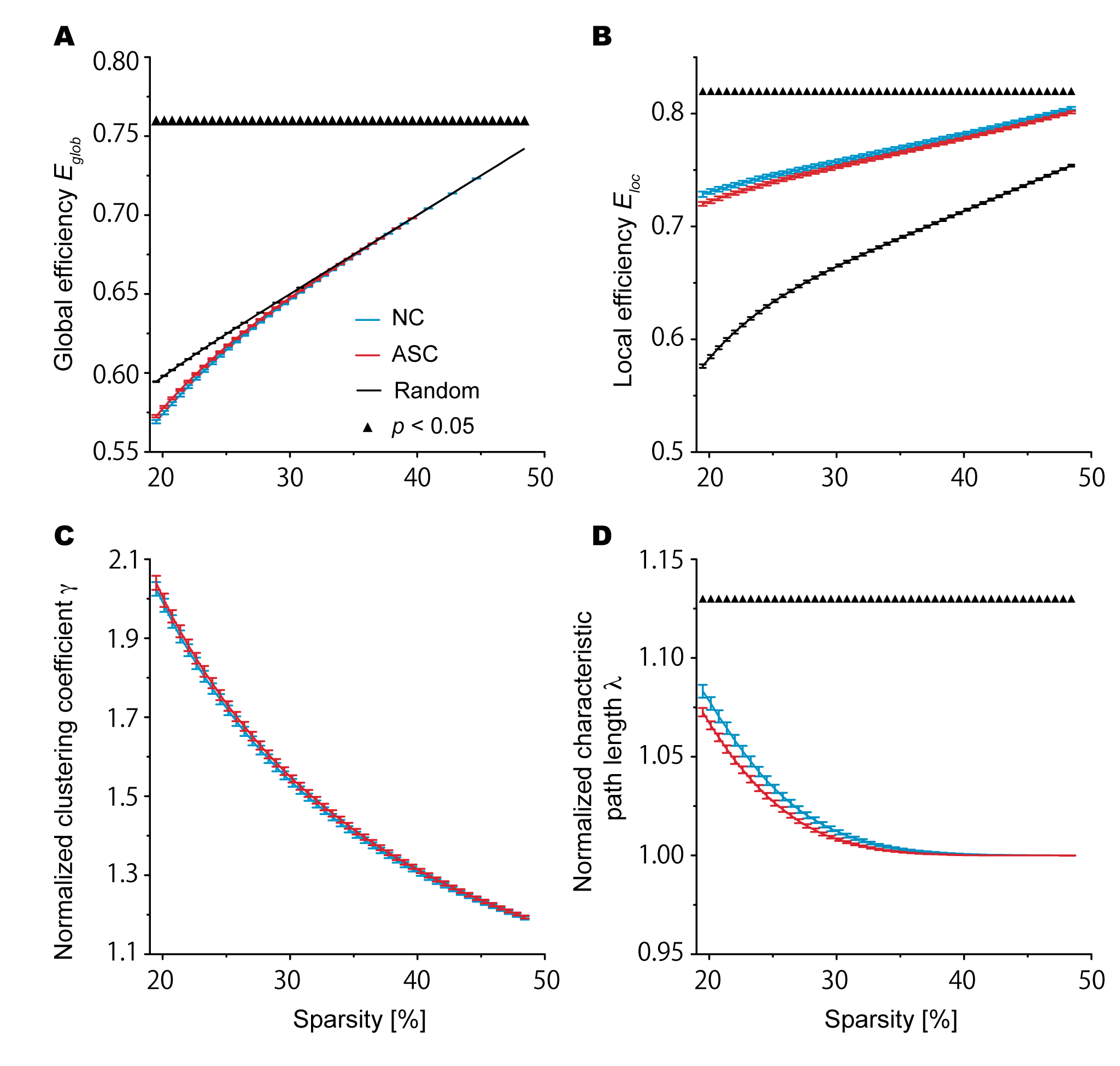

Supplement: Figure S2 — Global metrics of global efficiency, Eglob , local efficiency, Eloc , normalized clustering coefficient, γ, and normalized characteristic path length, λ, as functions of the sparsity threshold. The error bar indicates the standard error of the mean (SEM). Compared with the NC group (blue line), the ASC group (red line) showed significantly higher Eglob and lower Eloc and λ (p<0.05, FDR corrected) over the range of sparsity thresholds (A, B, and D), whereas γ was comparable between the groups (C). (TIF) [file pone.0094115.s002.tif]

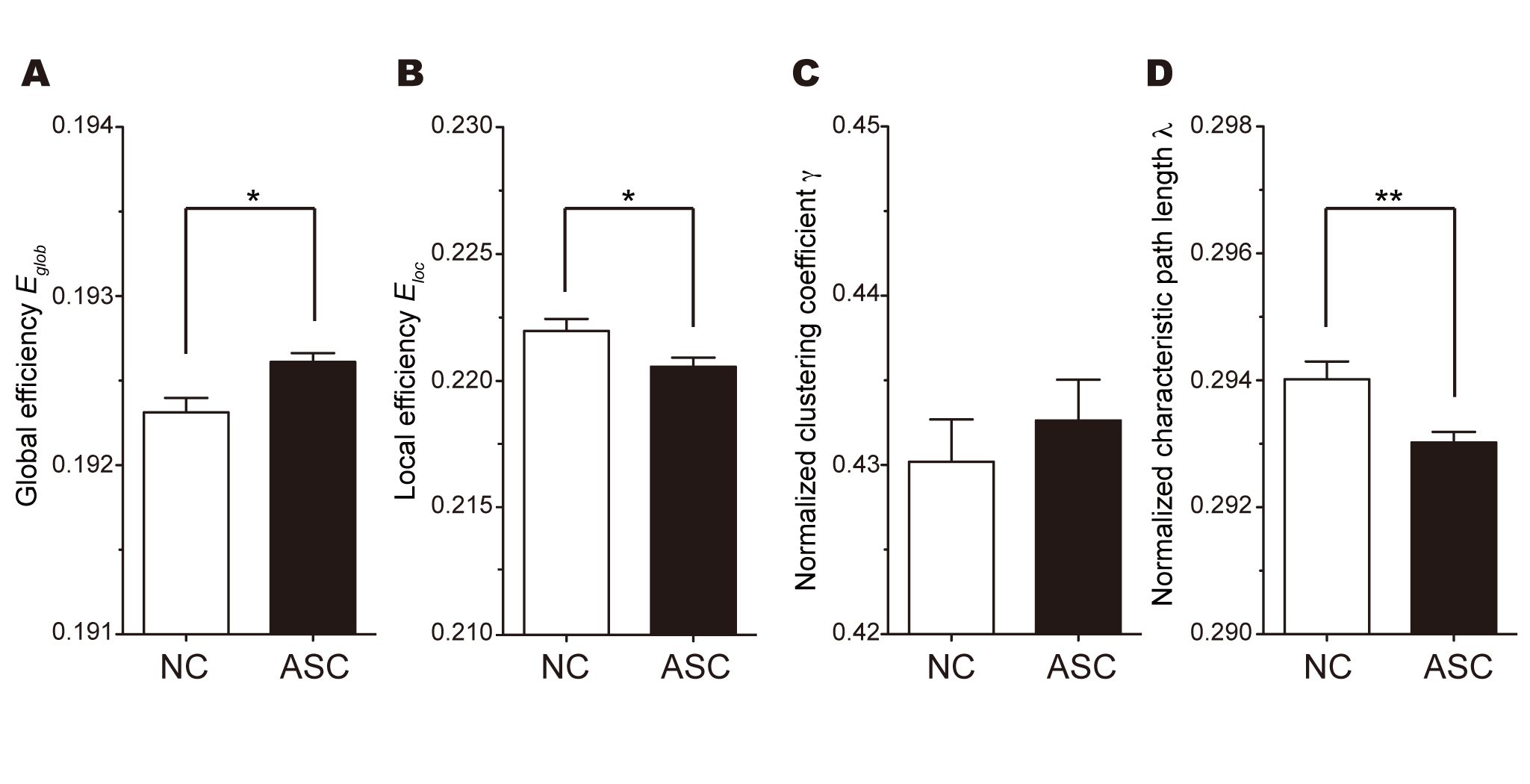

Supplement: Figure S3 — Between-group differences in the AUC values of global efficiency, Eglob , local efficiency, Eloc , normalized clustering coefficient, γ, and normalized characteristic path length, λ. In the AUC analyses, participants with ASC (black) exhibited significantly higher Eglob (p = 0.02) (A), and significantly lower Eloc (p = 0.011) (B) and λ (p = 0.001) (D), while γ was comparable between the groups (D). Significance levels are represented by *p<0.05 and **p<0.01, respectively. (TIF) [file pone.0094115.s003.tif]

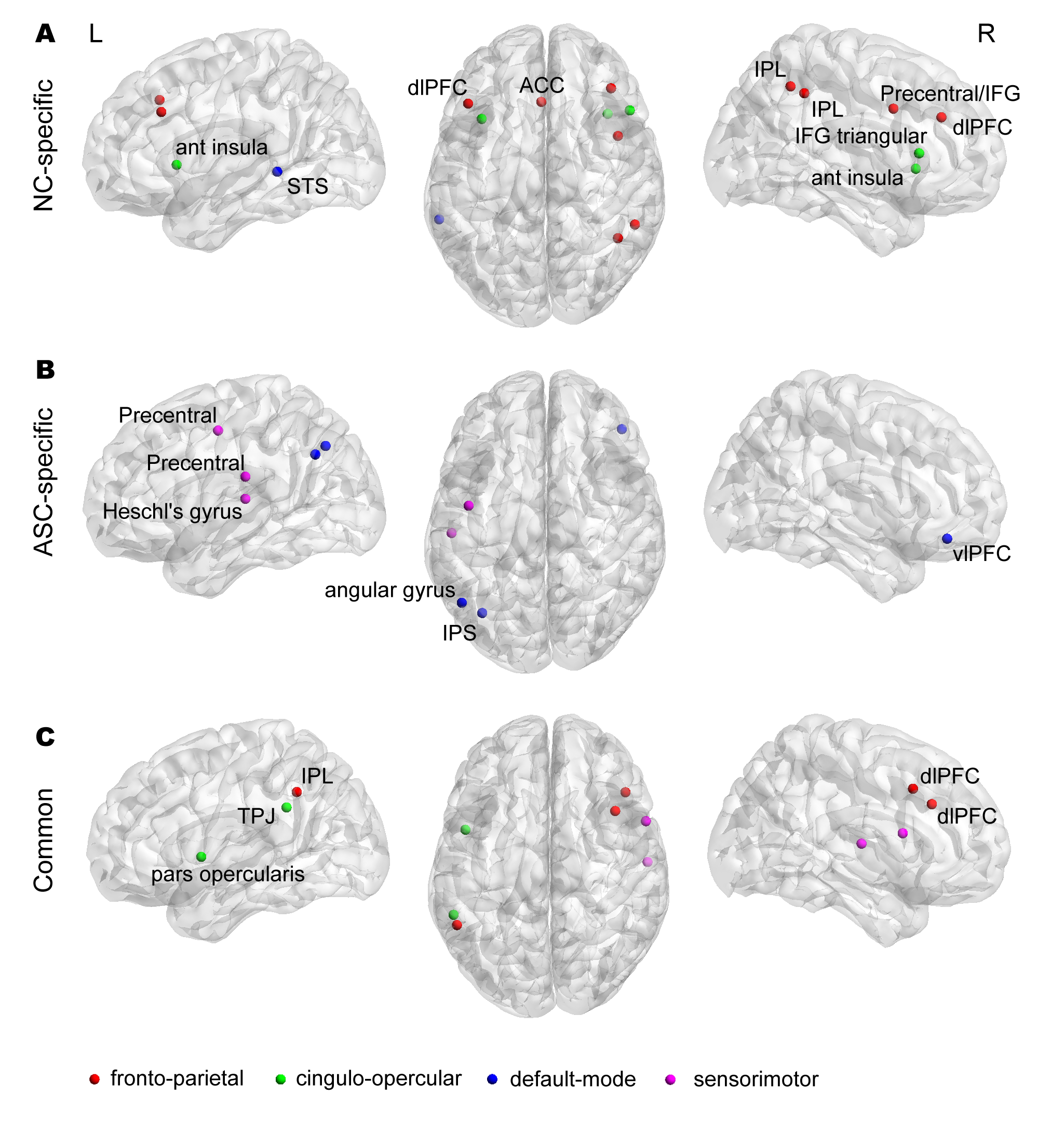

Supplement: Figure S4 — Functional hubs were identified using degree and bootstrapping method. The first row shows ten “NC-specific” hubs, involving the bilateral anterior insula, left STS, the bilateral dlPFC, and right IFG triangular; the second row shows six “ASC-specific” hubs, including the right vlPFC, left precentral gyrus, and left Heschl’s gyrus; and the last row shows five “common” hubs (the right dlPFC, left IPL, TPJ, and left IFG pars opercularis). Correspondences between colors and networks are as follows: fronto-parietal = red; cingulo-opercular = green; default mode = blue; sensorimotor = magenta. Hubs were visualized with the BrainNet Viewer (http://www.nitrc.org/projects/bnv/). (TIF) [file pone.0094115.s004.tif]

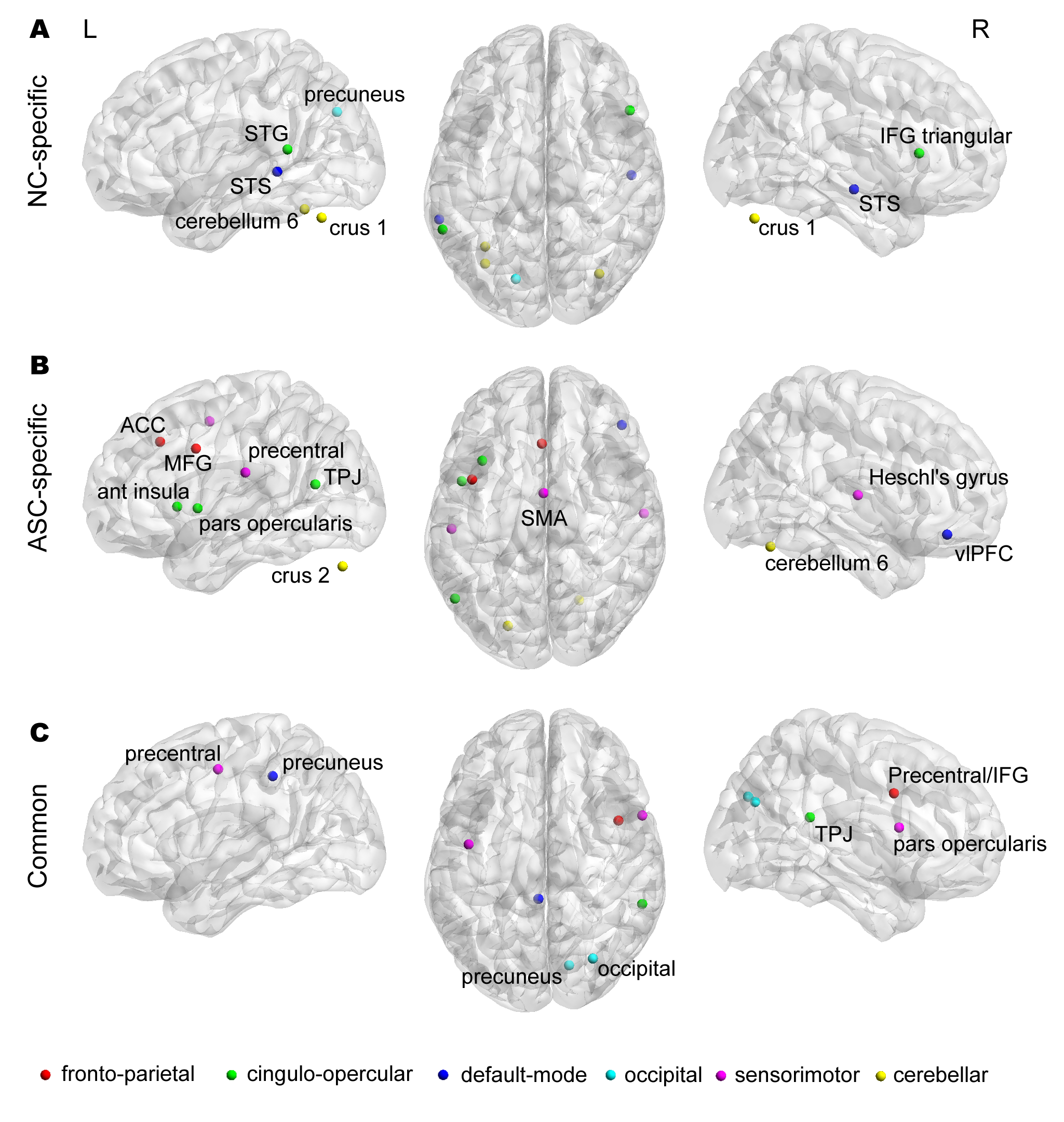

Supplement: Figure S5 — Functional hubs were identified using betweenness and bootstrapping method. The first row shows eight “NC-specific” hubs, involving the bilateral STS, left precuneus, the bilateral crus 1, and right IFG triangular; the second row shows 11 “ASC-specific” hubs, including the SMA, the right vlPFC, left TPJ, and right Heschl’s gyrus; and the last row shows seven “common” hub, encompassing the bilateral precuneus, the right TPJ, the right IFG pars opercularis, and left precentral. Correspondences between colors and networks are as follows: fronto-parietal = red; cingulo-opercular = green; default mode = blue; occipital = cyan; sensorimotor = magenta; cerebellar = yellow. Hubs were visualized with the BrainNet Viewer (http://www.nitrc.org/projects/bnv/). (TIF) [file pone.0094115.s005.tif]

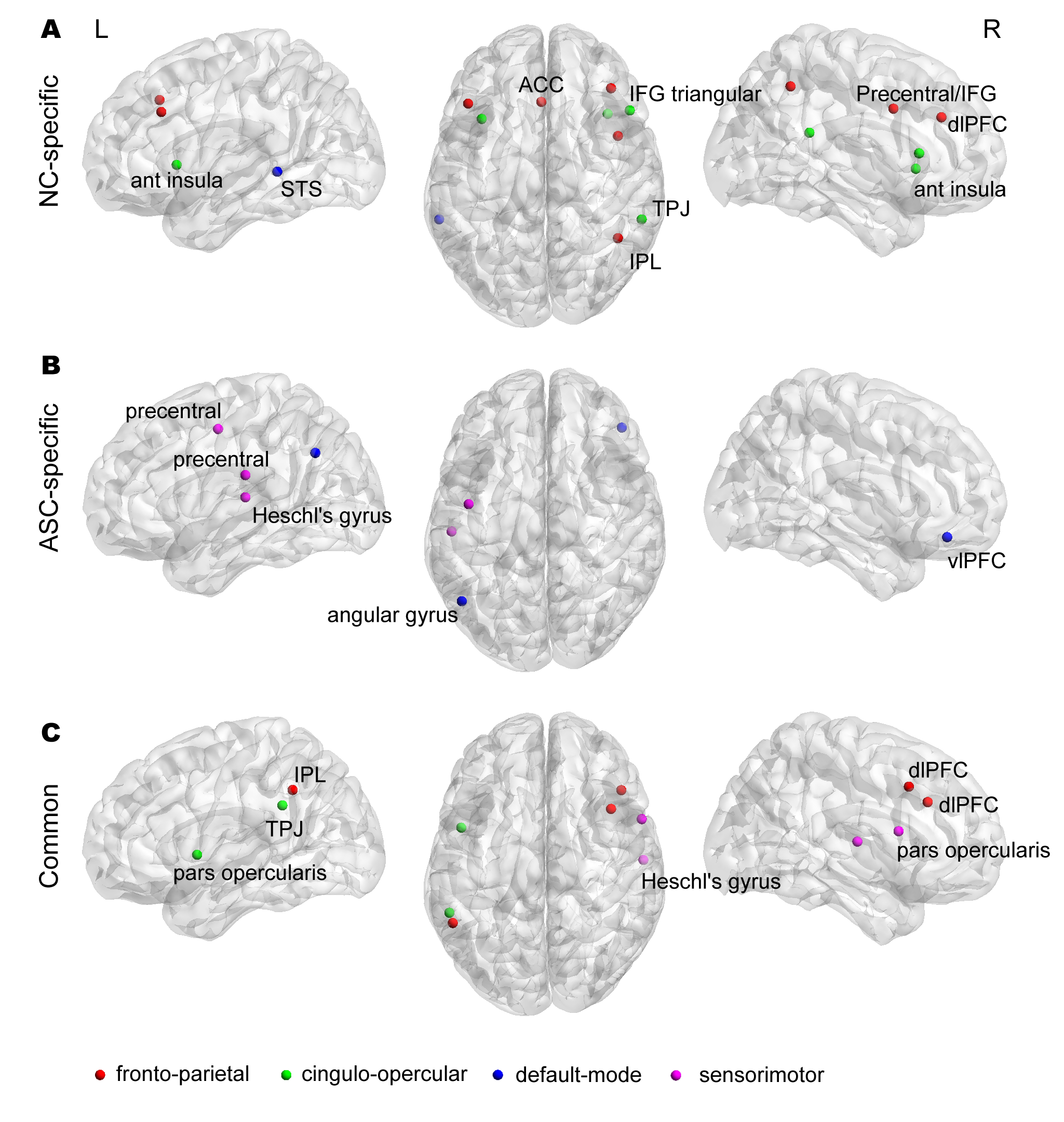

Supplement: Figure S6 — Functional hubs were identified using nodal efficiency and bootstrapping method. The first row shows nine “NC-specific” hubs, involving the bilateral anterior insula, the left STS, the left ACC, the right dlPFC, and right IFG triangular; the second row shows five “ASC-specific” hubs, including the right vlPFC, left precentral, left angular gyrus, and left Heschl’s gyrus; and the last row shows seven “common” hub, encompassing the bilateral IFG pars opercularis, and right dlPFC. Correspondences between colors and networks are as follows: fronto-parietal = red; cingulo-opercular = green; default mode = blue; sensorimotor = magenta. Hubs were visualized with the BrainNet Viewer (http://www.nitrc.org/projects/bnv/). (TIF) [file pone.0094115.s006.tif]

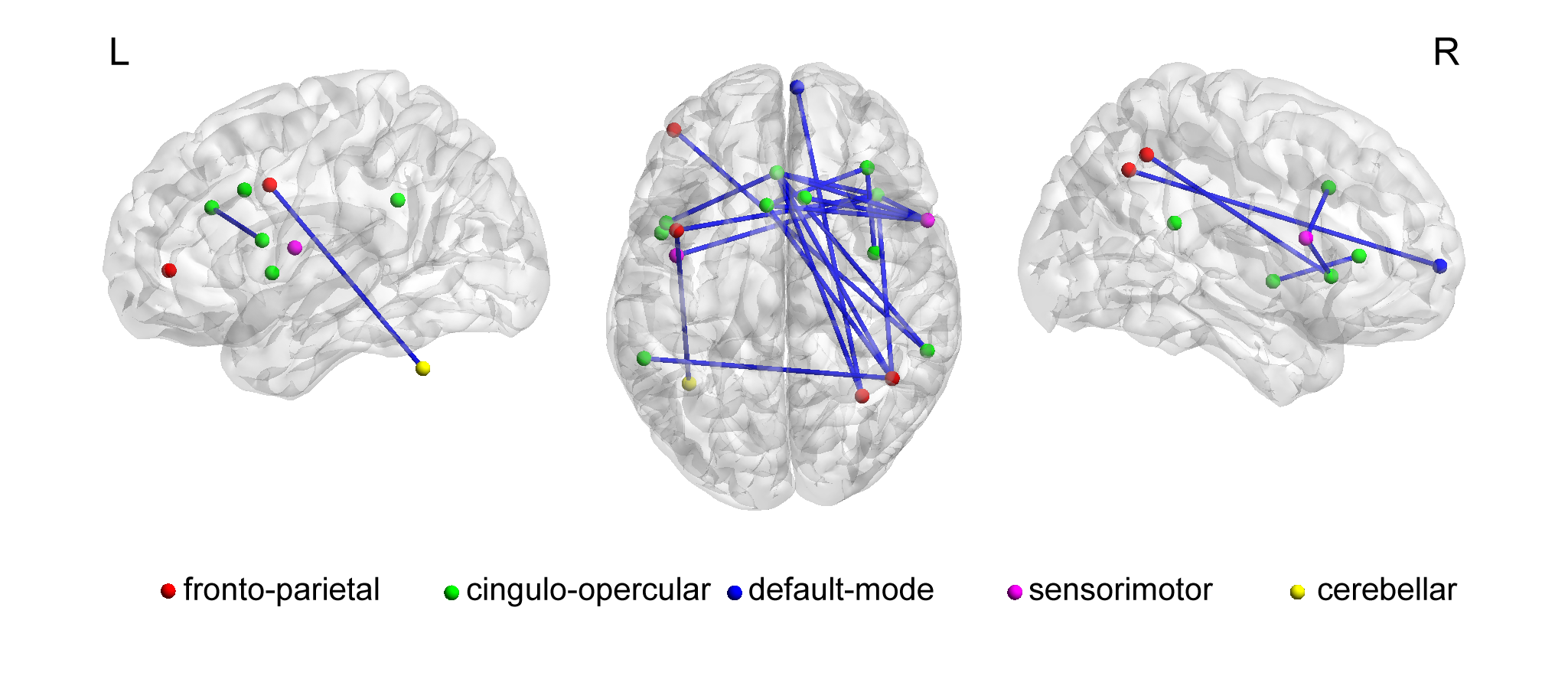

Supplement: Figure S7 — Reduced functional connectivity in participants with ASC identified using the network-based statistic approach. The reduced connections were found mainly within the cingulo-opercular network rather than within the default mode network. Correspondences between colors and networks are as follows: fronto-parietal = red; cingulo-opercular = green; default mode = blue; sensorimotor = magenta; cerebellar = yellow. These reduced connections were visualized with the BrainNet Viewer (http://www.nitrc.org/projects/bnv/). (TIF) [file pone.0094115.s007.tif]

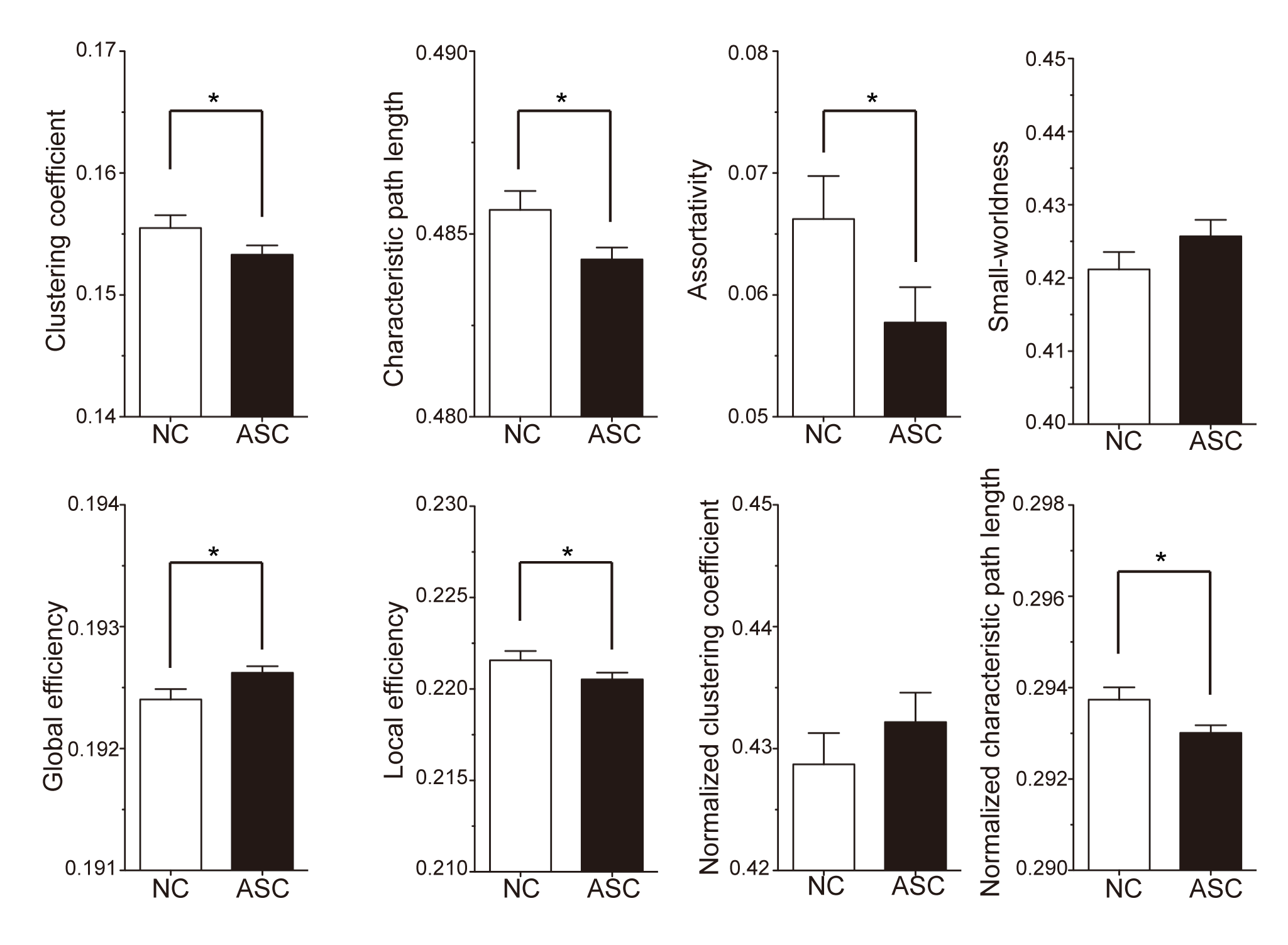

Supplement: Figure S8 — Between group differences in the AUC values of all the global measures performed on 39 NCs and 45 participants with ASC. The second-level analyses were re-conducted on 39 NCs and 45 ASC participants to ensure that between-group differences were not due to differences in handedness. Statistical conclusions were preserved for all the global metrics (clustering coefficient: p = 0.048, characteristic path length: p = 0.012; assortativity: p = 0.032; small-worldness: p = 0.083; global efficiency: p = 0.018; local efficiency: p = 0.044; normalized clustering coefficient: p = 0.170; normalized characteristic path length: p = 0.013). Significance levels are represented by *p<0.05 and **p<0.01, respectively. (TIF) [file pone.0094115.s008.tif]
