# Supplementary material for: Altered Network Topologies and Hub Organization in Adults with Autism: A Resting-State fMRI Study
Source: PLoS One. 2014 Apr 8;9(4):e94115. doi: 10.1371/journal.pone.0094115 (PMC3979738; doi:10.1371/journal.pone.0094115)
Supplement: Table S1 — Brief description of network metrics used in this study. (DOC) [file pone.0094115.s009.doc]

Table S1: Brief description of network metrics used in this study.

| **Network metric** | **Symbol** | **Description** |
| --- | --- | --- |
| **Global metrics** |  |  |
| Global efficiency | *E_glob_* | Global efficiency of a network quantifies the network’s capacity for parallel information transfer between nodes via multiple series of edges. |
| Local efficiency | *E_loc_* | Local efficiency of a network quantifies the ability of information transfer of the network at the local level, indicating the fault tolerance of the network. |
| Assortativity | *r* | Assortativity is a correlation coefficient between the degrees of all nodes on the two opposite ends of an edge. |
| Clustering coefficient | *C* | Clustering coefficient of a network measures how many local clusters exist in the network. |
| Characteristic path length | *L* | Characteristic path length of a network represents how well connected the network is. |
| Normalized clustering coefficient | *γ* | Normalized clustering coefficient is rescaled measure such that the original clustering coefficient of a network is divided by the averaged clustering coefficient of 100 random networks. |
| Normalized characteristic path length | *λ* | Normalized characteristic path length is normalized metric such that the original characteristic path length of a network is divided by the averaged characteristic path length of 100 random networks. |
| Small-worldness scalar | *σ* | Small-worldness scalar is calculated by *σ*=*γ*/*λ*. Small-world networks should satisfy *σ*>1. |
| **Local metrics** |  |  |
| Degree | *k* | Degree of a node quantifies the connectedness of the node with the rest of nodes in a network. |
| Betweenness | *b* | Betweenness of a node captures the influence of the node over information flow between all the other nodes in the network. |
| Nodal efficiency | *e* | Nodal efficiency of a node quantifies the ability of information propagation of the node with the rest of nodes in a network. |
| **Hub disruption index** | *κ* | Hub disruption index captures the pattern of abnormal nodal changes in patients against normative network topology using a local metric. |
