# Supplementary material for: Altered Network Topologies and Hub Organization in Adults with Autism: A Resting-State fMRI Study
Source: PLoS One. 2014 Apr 8;9(4):e94115. doi: 10.1371/journal.pone.0094115 (PMC3979738; doi:10.1371/journal.pone.0094115)
Supplement: Table S2 — Altered local network metrics (degree k , betweenness b , and nodal efficiency e ) in the ASC group compared to the NC group. (DOC) [file pone.0094115.s010.doc]

Table S2: Altered local network metrics (degree *k*, betweenness *b*, and nodal efficiency *e*) in the ASC group compared to the NC group.

| **Nodes** | **Coordinate** | **Type** | ***p* value** | | |
| --- | --- | --- | --- | --- | --- |
|  |  |  | *k* | *b* | *e* |
| **ASC>NC** |  |  |  |  |  |
| MFG orbital part R | 42, 48, -3 | FP | n.s. | 0.035 | n.s. |
| TPJ L | -52, -63, 15 | CO | 0.034 | n.s. | 0.045 |
| pars opercularis L | -46, -10, 14 | CO | n.s. | 0.038 | n.s. |
| precuneus R | 5, -50, 33 | DEF | 0.045 | 0.028 | n.s. |
| vlPFC R | 46, 39, -15 | DEF | n.s. | 0.022 | n.s. |
| postoccipital R | 33, -81, -2 | OC | 0.039 | n.s. | 0.032 |
| postoccipital L | -37, -83, -2 | OC | 0.033 | n.s. | 0.023 |
| occipital R | 9, -76, 14 | OC | n.s. | 0.008 | n.s. |
| occipital R | 20, -78, -2 | OC | n.s. | 0.033 | n.s. |
| postoccipital L | -5, 80, 9 | OC | n.s. | 0.043 | n.s. |
| SMA | 0, -1, 52 | SE | 0.031 | 0.006 | 0.046 |
| precentral L | -38, -15, 59 | SE | n.s. | 0.008 | n.s. |
| postcentral R | 46, -20, 45 | SE | n.s. | 0.048 | n.s. |
| **NC>ASC** |  |  |  |  |  |
| dlPFC R | 40, 36, 29 | FP | 0.023 | n.s. | 0.023 |
| IFG triangular L | -52, 28, 17 | FP | 0.024 | n.s. | 0.023 |
| IPL L | -35, -46, 48 | FP | n.s. | 0.040 | n.s. |
| IPL R | 44, -52, 47 | FP | 0.009 | n.s. | 0.009 |
| vPFC R | 34, 32, 7 | CO | 0.028 | n.s. | 0.026 |
| ACC L | -2, 30, 27 | CO | 0.026 | 0.049 | 0.025 |
| IFG trinagular R | 51, 23, 8 | CO | 0.006 | n.s. | 0.006 |
| fusiform R | 54, -31, -18 | CO | 0.008 | n.s. | 0.008 |
| STS R | 52, -15, -13 | DEF | 0.045 | 0.031 | 0.040 |
| fusiform R | 28, -37, -15 | DEF | n.s. | 0.028 | n.s. |
| midinsula R | 33, -12, 16 | SE | n.s. | 0.023 | n.s. |
| supramarginal L | -41, -31, 48 | SE | n.s. | 0.002 | n.s. |
| cerebellum 6 L | -24, -54, -21 | CER | n.s. | 0.033 | n.s. |
| vermis 8 R | 32, -61, -31 | CER | n.s. | 0.034 | n.s. |
| crus 1 R | 33, -73, -30 | CER | n.s. | 0.008 | n.s. |

ASC: autism spectrum condition, NC: normal control, PFC: prefrontal cortex, TPJ: temporoparietal junction, vlPFC: ventrolateral PFC, SMA: supplementary motor area, dlPFC: dorsolateral PFC, IPL: inferior parietal lobule, vPFC: ventral PFC, ACC: anterior cingulate cortex, STS: superior temporal sulcus, FP: fronto-parietal, CO: cingulo-opercular, DEF: default mode, OC: occipital, SE: sensorimotor, CER: cerebellar, n.s.: not significant, R: right, L: left.
