# Supplementary material for: Altered Network Topologies and Hub Organization in Adults with Autism: A Resting-State fMRI Study
Source: PLoS One. 2014 Apr 8;9(4):e94115. doi: 10.1371/journal.pone.0094115 (PMC3979738; doi:10.1371/journal.pone.0094115)
Supplement: Table S3 — A list of re-labeled nodes in this study. (DOC) [file pone.0094115.s011.doc]

Table S3: A list of re-labeled nodes in this study.

| **Label defined originally by Dosenbach et al.** | **Coordinate** | **Hemisphere** | **Type** | **Label used in this study** | **BA** |
| --- | --- | --- | --- | --- | --- |
| vPFC | -52, 28, 17 | L | FP | IFG triangular | - |
| postparietal | -35, 46, 48 | L | FP | IPL | - |
| vFC | 51, 23, 8 | R | FP | IFG triangular | BA 45 |
| inftemporal | 52, -15, -13 | R | DEF | STS | - |
| inftemporal | -61, -41, -2 | L | DEF | STS | BA 21 |
| ventaPFC | 42, 48, -3 | R | DEF | OFC | - |
| frontal | 58, 11, 14 | R | SE | pars opercularis | - |
| dFC | -42, 7, 36 | L | SE | MFG | - |
| parietal | -38, -15, 59 | L | SE | precentral | - |
| parietal | 46, -20, 45 | R | SE | postcentral | - |
| postparietal | -41, -31, 48 | L | SE | supramarginal | - |
| mid insula | 37, -2, -3 | R | CO | putamen | - |
| basal ganglia | -6, 17, 34 | L | CO | pre-SMA/ACC | - |
| parietal | 58, -41, 20 | R | CO | TPJ | - |
| parietal | -55, -44, 30 | L | CO | TPJ | - |
| vFC | -46, 10, 14 | L | CO | pars opercularis | - |
| vFC | -48, 6, 1 | L | CO | pars opercularis | - |
| latcerebellum | -24, -54, -21 | L | CER | Cerebellum 6 | - |
| infcerebellum | 32, -61, -31 | R | CER | vermis 8 | - |
| infcerebellum | 33, -73, -30 | R | CER | crus 1 | - |
| infcerebellum | -37, -54, -37 | L | CER | crus 1 | - |

PFC: prefrontal cortex, TPJ: temporoparietal junction, vlPFC: ventrolateral PFC, SMA: supplementary motor area, dlPFC: dorsolateral PFC, IPL: inferior parietal lobule, vPFC: ventral PFC, ACC: anterior cingulate cortex, STS: superior temporal sulcus, FP: fronto-parietal, CO: cingulo-opercular, DEF: default mode, OC: occipital, SE: sensorimotor, CER: cerebellar, n.s.: not significant, R: right, L: left, BA: brodmann area.
