# Supplementary material for: Altered Network Topologies and Hub Organization in Adults with Autism: A Resting-State fMRI Study
Source: PLoS One. 2014 Apr 8;9(4):e94115. doi: 10.1371/journal.pone.0094115 (PMC3979738; doi:10.1371/journal.pone.0094115)
Supplement: Table S4 — Reduced functional connectivity in participants with ASC compared to NCs. (DOC) [file pone.0094115.s012.doc]

Table S4: Reduced functional connectivity in participants with ASC compared to NCs.

| **Node A** | **Coordinate** | **Type** | **Node B** | **Coordinate** | **Type** | ***D* [mm]** | ***t*-value** |
| --- | --- | --- | --- | --- | --- | --- | --- |
| ant insula R | 38, 21, -1 | CO | pars opercularis R | 58, 11, 14 | SE | 26.93 | 4.09 |
| ACC L | -2, 30, 27 | CO | pars opercularis R | 58, 11, 14 | SE | 64.27 | 3.97 |
| pre-SMA/ACC L | -6, 17, 34 | CO | pars opercularis R | 58, 11, 14 | SE | 67.32 | 3.93 |
| IPL R | 44, -52, 47 | FP | pre-SMA/ACC L | -6, 17, 34 | CO | 86.20 | 3.89 |
| ACC L | -2, 30, 27 | CO | TPJ R | 58, -41, 20 | CO | 93.22 | 3.66 |
| IPL R | 44, -52, 47 | FP | TPJ L | -55, -44, 30 | CO | 100.77 | 3.59 |
| dACC R | 9, 20, 34 | CO | pars opercularis R | 58, 11, 14 | SE | 53.68 | 3.51 |
| ant insula R | 38, 21, -1 | CO | post insula L | -42, -3, 11 | SE | 84.38 | 3.44 |
| ventaPFC L | -43, 47, 2 | FP | TPJ R | 58, -41, 20 | CO | 135.16 | 3.39 |
| IPL R | 44, -52, 47 | FP | ACC L | -2, 30, 27 | CO | 96.12 | 3.36 |
| vPFC R | 34, 32, 7 | CO | pre-SMA/ACC L | -6, 17, 34 | CO | 50.54 | 3.33 |
| ACC L | -2, 30, 27 | CO | ant insula R | 38, 21, -1 | CO | 49.65 | 3.31 |
| MFG L | -42, 7, 36 | SE | crus 1 L | -37, -54, -37 | CER | 95.26 | 3.30 |
| IPL R | 44, -52, 47 | FP | ant insula R | 38, 21, -1 | CO | 87.57 | 3.30 |
| ant insula R | 38, 21, -1 | CO | pars opercularis L | -48, 6, 1 | CO | 87.32 | 3.28 |
| vmPFC R | 6, 64, 3 | DEF | IPS R | 32, -59, 41 | FP | 131.34 | 3.22 |
| MFG L | -42, 7, 36 | FP | ant insula R | 38, 21, -1 | CO | 89.25 | 3.20 |
| ACC L | -2, 30, 27 | CO | pars operculari L | -46, 10, 14 | CO | 50.05 | 3.19 |
| vPFC R | 34, 32, 7 | CO | putamen R | 37, -2, -3 | CO | 35.57 | 3.13 |
| IPS R | 32, -59, 41 | FP | ACC L | -2, 30, 27 | CO | 96.30 | 3.12 |

Note: ASC: autism spectrum condition, NC: normal control, FP: fronto-parietal, CO: cingulo-opercular, DEF: default mode, OC: occipital, SE: sensorimotor, CER: cerebellar, R: right, L: left, *D*: the Euclidian distance between node A and node B, ACC: anterior cingulate cortex, IPL: inferior parietal lobule, PFC: prefrontal cortex, FC: frontal cortex, IPS: intraparietal sulcus.
