# Supplementary material for: Altered Network Topologies and Hub Organization in Adults with Autism: A Resting-State fMRI Study
Source: PLoS One. 2014 Apr 8;9(4):e94115. doi: 10.1371/journal.pone.0094115 (PMC3979738; doi:10.1371/journal.pone.0094115)
Supplement: Text S1 — The details of scrubbing method used in this study. (DOC) [file pone.0094115.s013.doc]

**Text S1: The details of scrubbing method used in this study.**

We adopted the scrubbing method in the following order [1]: 1) the “motion-contaminated” volumes were detected using a frame-wise displacement (FD) threshold of 0.5 mm [2]; 2) the signal values of the motion-contaminated volumes were interpolated by applying the cubic spline function; 3) a band-pass filter (0.009–0.08 Hz) was then applied in order to reduce the effects of low-frequency drifts and high-frequency physiological noises; 4) finally, the interpolated volumes were deleted. The number of retained volumes was comparable between the groups (NC: 203.17 ± 1.51 volumes; ASC; 202.93 ± 2.51 volumes; *t*-test: *t* = 0.554, *p* = 0.581). The effect of this scrubbing method on correlation matrix is depicted in Figure S1.

**Supplementary References**

1. Carp J (2013) Optimizing the order of operations for movement scrubbing: Comment on Power et al. Neuroimage 76: 436-438.

2. Power JD, Barnes KA, Snyder AZ, Schlaggar BL, Petersen SE (2012) Spurious but systematic correlations in functional connectivity MRI networks arise from subject motion. Neuroimage 59: 2142-2154.
