# Supplementary material for: Altered Network Topologies and Hub Organization in Adults with Autism: A Resting-State fMRI Study
Source: PLoS One. 2014 Apr 8;9(4):e94115. doi: 10.1371/journal.pone.0094115 (PMC3979738; doi:10.1371/journal.pone.0094115)
Supplement: Text S2 — Functional connectivity analysis. (DOC) [file pone.0094115.s014.doc]

**Text S2: Functional Connectivity Analysis.**

**Statistical Analysis of Functional Connectivity**

We aimed to identify significantly altered functional connectivity in participants with ASC compared with NCs. The network-based statistic (NBS) approach was used for this purpose [1]. The NBS is a method for identifying connections in a graph while controlling the family-wise error rate in mass-univariate testing. Briefly, the NBS approach was performed as follows: first, the *t*-statistic was computed independently for each edge using a primary threshold (*t* = 3.1) to construct a set of supra-threshold edges. Then, all connected components in the set of supra-threshold edges were identified using a breadth first search, and the number of edges and their component size were determined. The null distribution of the maximal component size was derived from permutation tests, with 5000 repetitions. In each permutation, all participants were randomly allocated to one of the two groups, while keeping the number of participants in each group the same as in the original samples. Next, the *t*-statistic was calculated, after which the same threshold was applied to define a set of supra-threshold edges. Finally, for a connected component of size *M* found in the right grouping of NC and ASC, the corrected *p*-value was determined by finding the proportion of the 5000 permutations for which the maximal component was larger than *M*. Age and sex were included as nuisance covariates in the NBS approach.

**Results of Functional Connectivity Analyses**

The results of the NBS are shown in Figure S7 and Table S4. We found that 20 pairs of nodes showed significantly decreased connectivity in the ASC group, and no pair of nodes showed stronger connectivity (*p* = 0.030, corrected). With the exception of two pairs, all other node pairs were found either within the cingulo-opercular (CO) network (e.g., the connection between the anterior cingulate cortex (ACC) and right parietal cortex), or between the CO and other networks (e.g., the connection between the ACC and right frontal cortex in the sensorimotor network).

**Supplementary References**

1. Zalesky A, Fornito A, Bullmore ET (2010) Network-based statistic: identifying differences in brain networks. Neuroimage 53: 1197-1207.
